# General Post‐Regulation Strategy of AIEgens’ Photophysical Properties for Intravital Two‐Photon Fluorescence Imaging

**DOI:** 10.1002/advs.202404792

**Published:** 2024-08-09

**Authors:** Liyun Lin, Jiaxin Liu, Zhengyuan Pan, Wen Pang, Xinyan Jiang, Man Lei, Jucai Gao, Yujie Xiao, Bo Li, Fang Hu, Zhouzhou Bao, Xunbin Wei, Wenbo Wu, Bobo Gu

**Affiliations:** ^1^ School of Biomedical Engineering Shanghai Jiao Tong University Shanghai 200030 China; ^2^ Department of Chemistry Institute of Molecular Aggregation Science Tianjin University Tianjin 300072 China; ^3^ Biomaterials Research Center School of Biomedical Engineering Southern Medical University Guangzhou 510515 China; ^4^ Department of Neurology Huashan Hospital MOE Frontiers Center for Brain Science State Key Laboratory of Medical Neurobiology Institutes for Translational Brain Research Fudan University Shanghai 200437 China; ^5^ Shanghai Key Laboratory of Gynecologic Oncology Ren Ji Hospital School of Medicine Shanghai Jiao Tong University Shanghai 200127 China; ^6^ Laboratory of Carcinogenesis and Translational Research (Ministry of Education/Beijing) Peking University Cancer Hospital & Institute Beijing 100142 China; ^7^ Biomedical Engineering Department and International Cancer Institute Peking University Beijing 100191 China

**Keywords:** aggregation‐induced emission, general post‐regulation, two‐photon fluorescence imaging

## Abstract

Fluorogens with aggregation‐induced emission (AIEgens) are promising agents for two‐photon fluorescence (TPF) imaging. However, AIEgens’ photophysical properties are fixed and unoptimizable once synthesized. Therefore, it is urgent and meaningful to explore an efficient post‐regulation strategy to optimize AIEgens’ photophysical properties. Herein, a general and efficient post‐regulation strategy is reported. By simply tuning the ratio of inert AIEgens within binary nanoparticles (BNPs), the fluorescence quantum yield and two‐photon absorption cross‐section of functional AIEgens are enhanced by 8.7 and 5.4 times respectively, which are not achievable by conventional strategies, and the notorious phototoxicity is almost eliminated. The experimental results, theoretical simulation, and mechanism analysis demonstrated its feasibility and generality. The BNPs enabled deep cerebrovascular network imaging with ≈1.10 mm depth and metastatic cancer cell detection with single‐cell resolution. Furthermore, the TPF imaging quality is improved by the self‐supervised denoising algorithm. The proposed binary molecular post‐regulation strategy opened a new avenue to efficiently boost the AIEgens’ photophysical properties and consequently TPF imaging quality.

## Graphical abstract

A general post‐regulation approach is reported to improve AIEgens’ photophysical properties for intravital TPF imaging. This BNPs is composed of functional, inert AIEgens and encapsulation matrix (a). The functional AIEgens is a fluorescence component, and the inert AIEgens regulate the photophysical properties (b); The TPACCS is enhanced by 47‐fold, while the ROS is suppressed. The BNPs enable cerebrovascular network imaging with ≈1.10 mm depth and detection of metastatic cancer cells (c).

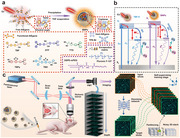

## 1 Introduction

Biomedical imaging methods, including X‐ray imaging, computer‐assisted tomography (CT), ultrasound imaging, magnetic resonance imaging (MRI), etc., count for a great deal in both fundamental research and clinical diagnosis, enabling improvements in healthcare quality.^[^ ^1^ ^]^ These medical imaging technologies mentioned above provide many distinguished advantages, but they also suffer from limited resolution. Fluorescence imaging is an indispensable platform due to its unique features, including high resolution at the subcellular level, strong signal with high signal‐to‐background (SBR), easy manipulation, etc.^[^ ^2^ ^]^ However, many reported fluorescence imaging methods suffer from shallow imaging depth since visible light undergoes strong scattering and absorption in highly heterogeneous biological tissue.^[^ ^1c^ ^]^ Considering that near‐infrared (NIR) light experiences reduced scattering and absorption,^[^ ^3^ ^]^ NIR‐light excited two‐photon fluorescence (TPF) imaging opens a new avenue to achieve high resolution at extended penetration depths, particularly significant for intravital imaging.^[^ ^4^ ^]^ However, conventional TPF agents were subjected to small two‐photon absorption cross‐sections (TPABCS, δ), low fluorescence quantum yields (FQY, η), and subsequently small two‐photon action cross‐sections (TPACCS, δη),^[^ ^5^ ^]^ inducing low SBR, extremely high power excitation, etc. The ideal TPF agent should possess high FQY and large TPABCS, thereby enabling high‐quality imaging at extended imaging depth. Recently, various emerging TPF agents with high FQY and large TPABCS, including organic fluorophores,^[^ ^6^ ^]^ semiconductor quantum dots,^[^ ^7^ ^]^ g‐C_3_N_4_ QDs,^[^ ^8^ ^]^ carbon dots,^[^ ^9^ ^]^ gold‐based nanoparticles (NPs),^[^ ^10^ ^]^ GFP chromophore analogs,^[^ ^11^ ^]^ fluorogens with aggregation‐induced emission (AIE) characteristics (AIEgens),^[^ ^12^ ^]^ have been designed and synthesized. AIEgens have various unique features such as flexible molecular structure, aggregation‐caused quenching (ACQ) resistance, etc., making it a promising TPF imaging agent.^[^ ^13^ ^]^ Moreover, AIEgens could achieve high FQY and large TPABCS by introducing donor‐acceptor (D‐A) structure,^[^ ^14^ ^]^ and the TPABCS could be further enhanced using extensive π‐conjugated molecules with strong D‐A interactions.^[^ ^15^ ^]^ However, the D‐A structure tends to induce phototoxicity under light excitation since the introduction of the D‐A structure could significantly decrease the energy gap (ΔE_ST_) between the singlet excitation energy and triplet excitation energy and thus increase the ^1^O_2_ generation effieicency,^[^ ^16^ ^]^ which is harmful to biological samples during performing bioimaging. Meanwhile, AIEgens’ photophysical properties are fixed once synthesized according to the design, the regulation and optimization of photophysical properties require iterative design and synthesis. Thus, it is urgent and meaningful to develop a simple and efficient strategy to post‐regulate AIEgens’ photophysical properties including FQY and TPABCS, both of which are key parameters to determine the TPF imaging quality. Recently, a doping method was proposed to enhance the FQY by controlling the energy relaxation pathway.^[^ ^17^ ^]^ However, the proposed methods could only enhance AIEgens’ FQY instead of both FQY and TPABCS. Therefore, rational and general post‐regulation strategies to enhance both FQY and TPABCS of AIEgens, which were accompanied with a high ROS inhibition rate and excellent biocompatibility, are extremely desirable for advancing TPF imaging.

**Scheme 1:**
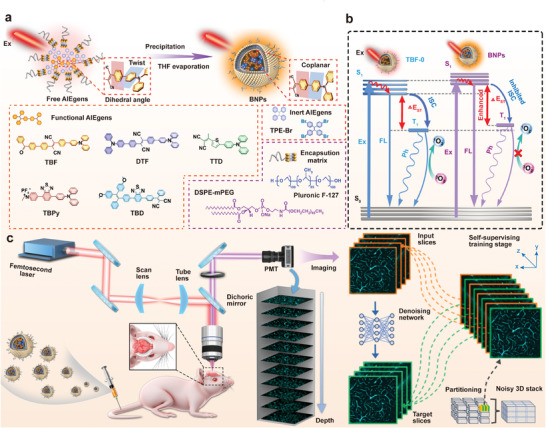
Scheme of the post‐regulation strategy and TPF imaging. a) The illustration of synthesis and working principle of BNPs and the chemical structures of functional AIEgens, inert AIEgens, and encapsulation matrix. b) The illustration of the energy relaxation of functional AIEgens (TBF as an example) before and after inert AIEgens regulation. c) The intravital TPF imaging of brain vasculature and image quality improvement by self‐supervised denoising algorithm. Ex: excitation; THF: tetrahydrofuran; TBF: 2‐(4′‐benzoyl‐[1,1′‐biphenyl]−4‐yl)−3‐(4′‐(diphenylamino)‐[1,1′‐biphenyl]−4‐yl)fumaronitrile; DTF: 2,3‐bis(4′‐(diphenylamino)‐[1,1′‐biphenyl]−4‐yl)fumaronitrile; TTD: 2‐(1‐(5‐(4‐(diphenylamino)phenyl)thiophen‐2‐yl)ethylidene)malononitrile; TBPy: 4‐(7‐(4‐(diphenylamino)phenyl)benzo[c][1,2,5]thiadiazol‐4‐yl)−1‐ethylpyridin‐1‐ium hexafluorophosphate; TBD: 2‐((4‐(7‐(4‐(2,2‐bis(4‐methoxyphenyl)−1‐phenylvinyl)phenyl)benzo[c][1,2,5]thiadiazol 4yl)phenyl)(phenyl)methylene) malononitrile; TPE‐Br: 1,1,2,2‐Tetrakis(4‐bromophenyl)ethylene. Pluronic F127: poly(ethylene oxide)‐b‐poly‐(propylene oxide)‐b‐poly(ethylene oxide). FL: fluorescence; Ph: phosphorescence; ISC: intersystem crossing; S_0_: the ground state; S_1_: the lowest singlet excited state; T_1_: the lowest triplet excited state; PMT: photomultiplier tube.

In this work, we reported a general approach to improve AIEgens’ photophysical properties for intravital TPF imaging (**Scheme** **1**). This approach relied on simply tuning the ratio of inert AIEgens within binary nanoparticles (BNPs), which were composed of three key components, i.e., functional AIEgens, inert AIEgens, and encapsulation matrix (Scheme 1a). Within BNPs, the functional AIEgens served as the fluorescent substance to absorb and convert photoenergy into fluorescence; the inert AIEgens served as regulatory molecules to regulate the dihedral angles, the surrounding polarity and ΔE_ST_ (singlet‐triplet energy gap) of the functional AIEgens (Scheme 1b); the encapsulation matrix acted as a support for transferring AIEgens from dispersed organic phase to nanoparticles. Compared with pure TBF nanoparticles, the FQY and TPABCS of TBF within BNPs were enhanced by 8.7 and 5.4 times, leading to a 47‐fold enhancement of TPACCS, while the ROS generation efficiency was suppressed to less than 1% of the original value. The mechanism, feasibility, and generality of post‐regulation strategy were experimentally demonstrated and theoretically analyzed. The optimized BNPs, with high FQY, large TPABCS, far‐red/NIR (FR/NIR) emission, excellent photostability, and superior biocompatibility, enabled cerebrovascular network imaging with ≈1.10 mm imaging depth and detection of metastatic cancer cells with single‐cell resolution under two‐photon excitation. Meanwhile, the imaging quality was further improved by a self‐supervised denoising algorithm.

## 2 Results

### 2.1 Photophysical Properties Characterization

TBF with AIE characteristics was designed and synthesized as building blocks for the study of the post‐regulation strategy. In our design, the D‐A structure of AIE active triphenylamine‐fumaronitrile was spaced by the π‐bridge of the phenyl ring and further linked a benzophenone moiety to the acceptor to enhance the spin‐orbit coupling between singlet and triplet states.^[^ ^18^ ^]^ Therefore, TBF was expected to possess FR/NIR emission with moderate FQY and TPABCS, and high ROS production, making it an optimal choice for studying our post‐regulation strategy in enhancing FQY and TPABCS as well as suppressing ROS production. The synthetic route to TBF is presented in Figure S1 (Supporting Information), and the chemical structure of TBF and the key intermediates were identified by NMR and high‐resolution mass spectra (Scheme 1 and Figures S2–S8, Supporting Information).

**Figure 1:**
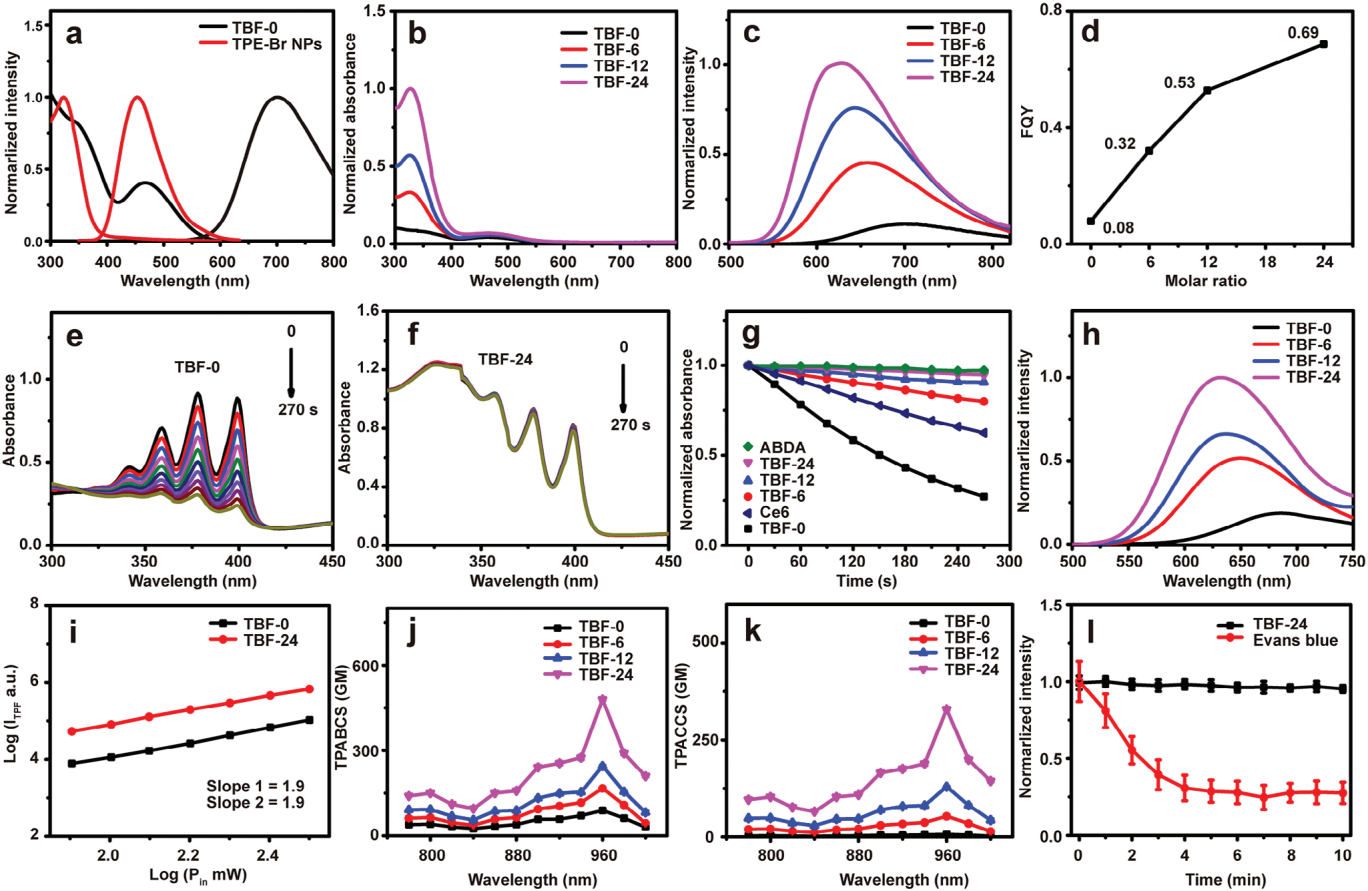
Photophysical properties. a) Normalized UV‐vis absorption and emission spectra of TBF‐0 (Ex: 467 nm) and TPE‐Br NPs (Ex: 320 nm). b) The absorption spectra, c) emission spectra, and d) the FQY of BNPs with different binary molecular ratios. The absorption spectra of the e) ABDA and TBF‐0, f) ABDA and TBF‐24 mixture solution under light irradiation for different times. g) The decomposition of ABDA at 399 nm in the presence of TBF‐0, TBF‐6, TBF‐12, TBF‐24, and Ce6 respectively under light irradiation at different times. [TBF‐0] = [BNPs] = 6.5 µM based on TBF, [Ce6] = 6.5 µM, [ABDA] = 50 µM, light irradiation: 400–700 nm, 70 mW cm^−2^. h) TPF spectra of BNPs with different binary molecular ratios, Ex: 960 nm, [BNPs] = 200 µM based on TBF. i) The dependence of TPF intensity (I_TPF_) on the excitation power (P_in_), Ex: 960 nm, [BNPs] = 200 µM based on TBF. The j) TPABCS and k) TPACCS of TBF‐0, TBF‐6, TBF‐12, and TBF‐24 at different wavelengths in aqueous solutions. l) Photostability of TBF‐24 and Evans blue under two‐photon excitation, Ex: 960 nm, E_Power_ = 30 mW.

TBF exhibited an absorption peak at 445 nm, while its emission in THF was negligible (Figure S9a,b, Supporting Information). Once adding water to its THF solution until the ratio of water to THF (*f*_w_) reached 60% (v/v), an emission centered at 638 nm was observed, demonstrating its AIE characteristics (Figure S9b, Supporting Information). The AIE phenomenon was also observed for TPE‐Br (Scheme 1 and Figure S9c, Supporting Information), which was selected as the inert AIEgens of BNPs for the following reasons: (I) TPE‐Br NPs (TPE‐Br nanoparticles: TPE‐Br encapsulated with PEGylated phospholipid) have the absorption peak located at 325 nm with tail extended to 420 nm, which will not be interfered by 467 nm laser used for excitation of functional TBF molecules (**Figure** **1**a); (II) TPE‐Br is a nonpolar molecule (dipole moment, 0.0001D), and the exist halogen‐halogen interactions could form a rigid microenvironment to enhance the intramolecular motion restriction of phenyl rings and consequently the FQY for the isolated TBF.

The synthesized TBF was mixed with TPE‐Br to form BNPs using PEGylated phospholipid (DSPE‐mPEG) as the encapsulation polymer by the coprecipitation method (Scheme 1a). When the binary molecular ratio (TBF to TPE‐Br by keeping TBF constant) changed from 1:0 (TBF‐0) to 1:6 (TBF‐6), 1:12 (TBF‐12), and 1:24 (TBF‐24), the BNPs had size range about 46~69 nm (Figure S10), Supporting Information. And these BNPs exhibited superior long‐term stability (Figure S11a,b, Supporting Information), and excellent stability in DMEM with 10% FBS (Figure S11c, Supporting Information), which was a body fluid‐mimicking environment. It seemed that the absorption spectra of BNPs were the spectral superposition of TBF and TPE‐Br (Figure 1b), indicating that the TPE‐Br had no influence on the absorption features of TBF. Once performing excitation at 467 nm, which could only excite TBF but not TPE‐Br, the emission peaks of BNPs, with binary molecular ratio changed from 1:0 to 1:24, were correspondingly changed from 702 nm to 658, 644, and 628 nm (Figure 1c), which were accompanied with the increment of FQY from 0.08 to 0.32, 0.53 and 0.69 (Figure 1d) using Rhodamine 6G (*η* = 0.95 in ethanol) as the reference.^[^ ^19^ ^]^ Although its FQY was further increased, TBF‐36 suffered from the instability (Figure S11d, Supporting Information), thus TBF‐24 was selected for the following studies. Due to the inevitable phototoxicity endowed by the introduction of the D‐A structure, the ROS generation efficiency of TBF‐0 was studied using 9,10‐anthracenediyl‐bis(methylene)dimalonic acid (ABDA) as an indicator. It was demonstrated that TBF‐0 had much better ROS generation capability than Ce6, one commercial photosensitizer (Figure 1e,f; Figure S12, Supporting Information). Then, the ROS generation efficiency of BNPs was also characterized in the same conditions and compared with TBF‐0. The decomposition of ABDA in the presence of TBF‐24 was far less than that in the presence of TBF‐0 (Figure 1e–g), indicating that the ROS generation capability of TBF‐0 and subsequently its phototoxicity were remarkably inhibited. TBF‐24 was efficiently regulated to be a non‐phototoxic fluorescence imaging agent with high FQY by simple post‐regulation, eliminating the troublesome repetitions of synthesis and modification.

The TPF spectra of BNPs were collected to analyze the optical nonlinear features (Figure 1h). TBF‐24, with the strongest TPF intensity among all BNPs, was selected for the following studies. A linear relationship with a slope of ≈2 between log_10_(input power) and log_10_(output power) was obtained for both TBF‐0 and TBF‐24 (Figure 1i), confirming two‐photon excitation of TBF within BNPs. To quantify the two‐photon excitation efficiency, the TPABCS was measured via a two‐photon induced fluorescence method^[^ ^20^ ^]^ using a home‐built fluorescence measuring system (Figure S13, Supporting Information). The measured TPABCS of TBF‐0 was 89.1 GM at 960 nm (Figure 1j), while the TPABCS of TBF‐24 was enhanced 5.4 times to 479.0 GM. Then, the TPACCS of TBF‐0 and BNPs were calculated at various wavelengths for comparison (Figure 1k). The TPACCS of TBF‐24 at 960 nm was 328.9 GM, which was enhanced 47 times as compared with that of TBF‐0. The significantly enhanced TPACCS, which could endow high SBR of TPF imaging even under low excitation power, was highly desirable for high‐quality bioimaging.

Photostability is of great importance for maintaining bright and stable TPF signals for long‐term and dynamic imaging. The photostability of TBF‐24 was examined and compared with the commercially available blood vascular imaging dye, i.e., Evans blue,^[^ ^21^ ^]^ which was regarded as one stable commercial fluorescent agent^[^ ^21^, ^22^ ^]^ and had a similar emission spectrum as that of TBF‐24 (Figure S14, Supporting Information). The TPF intensity of Evans blue decreased to 28% of the original intensity after 10 min continuous excitation (Figure 1l), while that of TBF‐24 remained ≈95%, indicating its excellent photostability under two‐photon excitation.

The experimental results presented above demonstrated that the post‐regulation efficiently improved the TPACCS of TBF and suppressed ROS production, making it a promising TPF agent. The TBF‐24 could efficiently light‐up HEK293, HeLa, and 143B cells under one‐photon and two‐photon excitation (Figure S15, Supporting Information), indicating good cellular internalization and imaging capabilities of TBF‐24.

### 2.2 Mechanism of Post‐Regulation

**Figure 2:**
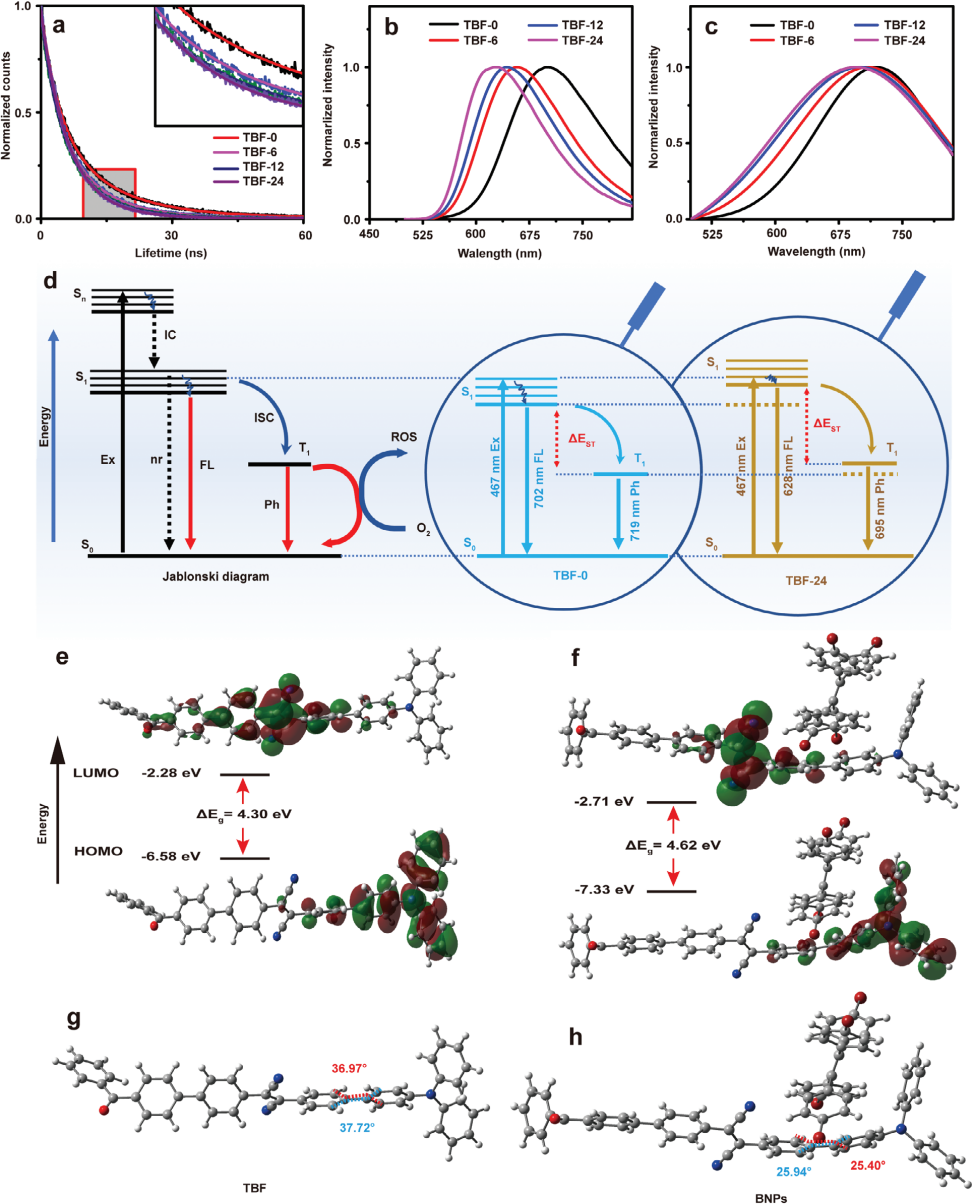
Experimental and theoretical investigation of post‐regulation mechanism. a) Fluorescence decay curves of BNPs. The inset is the enlarged part of the selected area. The lines denote the fitting curves. b) Fluorescence and c) phosphorescence spectra of BNPs, Ex: 467 nm. d) Schematic illustration of the energy levels for TBF‐0 and TBF‐24 and the dissipation pathways of excited energy. Optimized geometries and molecular orbital amplitude plots of HOMO–LUMO distribution of e) TBF and f) TBF in the presence of TPE‐Br calculated by TD‐DFT. The geometry optimizations of t1, t2, and t3 were carried out at the M06‐2X^1^/6‐311+g(d,p) level of theory using the Gaussian 09 software package. The dihedral angles of g) TBF and h) TBF in the presence of TPE‐Br. S_n_: the singlet excited state, T_1_: the lowest triplet excited state, nr: non‐radiative relaxation, Ex: excitation, IC: internal conversion, FL: fluorescence, Ph: phosphorescence.

The proposed post‐regulation strategy by simple binary molecular tuning is similar to diluting TBF using a nonpolar solvent. In solution, i.e., molecular states, TBF interacted with polar solvent molecules, resulting in red‐shifted and weakened fluorescence, and vice versa for a nonpolar solvent (Figure S16, Supporting Information). While in nanoparticles, i.e., aggregation states, polar TBF kept strong interaction with each other, restricting the FQY of TBF‐0. Once TPE‐Br was added to form BNPs, polar TBF (dipole moment, 3.9160 D) was separated by nonpolar TPE‐Br (dipole moment, 0.0001D), leading to a weaker dipole‐dipole interaction and increased FQY. In addition, the rigid TPE‐Br could also protect TBF away from strong polar water. The more TPE‐Br, the more chance for TBF to interact with nonpolar TPE‐Br, leading to the blue‐shifted emission and increased FQY since the surrounding polarity was decreased. It was well known that fluorogen with D‐A structures dissolved in less polar solvents could yield a shorter lifetime. Thus, the lifetime of TBF within different BNPs was studied, and the lifetimes of TBF changed from 11.11 to 8.27, 7.45, and 7.09 ns with an increment of binary molecular ratios (**Figure** **2**a). The experimental results were consistent with our deduction that TBF was separated further from each other to be less polar and consequently the lifetime of TBF became shorter. Thus, it could be concluded that the lifetime and FQY of TBF within BNPs were respectively shortened and increased with increment of binary molecular ratios once performing binary molecular tuning.

To analyze the post‐regulation mechanism, the fluorescence and phosphorescence spectra of BNPs were recorded. The emitted fluorescence (from S_1_ to S_0_) and phosphorescence (from T_1_ to S_0_) of TBF‐0 were located at 702 and 719 nm, respectively. When the binary molecular ratio was increased from 1:0 to 1:6, 1:12 and 1:24, the fluorescence emission peaks correspondingly shifted from 702 nm to 658, 644 and 628 nm (Figure 2b), which was consistent with the phenomenon that less polar surroundings induced blue‐shifted emission, meanwhile the phosphorescent emission peaks shifted from 719 nm to 709, 700 and 695 nm (Figure 2c). The shift of fluorescence emission (74 nm) was much larger than that of phosphorescent emission (24 nm), it could be deduced that Δ*E*_ST_, which indicated the efficiency of intersystem crossing (ISC) process from S_1_ to T_1_ and was one key factor to determine ROS generation,^[^ ^23^ ^]^ was increased (Figure 2d). The post‐regulation induced increment of Δ*E*_ST_ could significantly turn off the energy dissipation pathways of ISC process, and subsequently inhibit the generation of ROS as well as improve FQY.^[^ ^24^ ^]^

To further investigate the post‐regulation mechanism, the electronic distributions and molecular geometries of TBF were studied using density functional theory (DFT) calculations. Obvious overlap between HOMO and LUMO was observed for TBF with a relatively small Δ*E*_g_ of 4.30 eV (Figure 2e). While in the presence of TPE‐Br, the HOMO of TBF was almost totally localized over the triphenylamine group and the LUMO was primarily distributed over diphenyldinitrile ethylene. The effective separation of HOMO‐LUMO distribution led to an increased Δ*E*_g_ value of 4.62 eV (Figure 2f). The calculation results matched well with the spectral response. The dihedral angles were calculated before and after binary molecular tuning to study the enhancement mechanism of TPABCS (Figure 2g). The dihedral angles between triphenylamine and diphenyldinitrile ethylene in TBF were 36.97° and 37.72°, respectively. Once performing binary molecular regulation, the dihedral angles decreased to 25.40° and 25.97°, respectively (Figure 2h). The decreased distortion angles indicated that intramolecular D‐A conformation changed from twisted to coplanar one (Scheme 1a, Figure 2g,h), which was in favor of yielding better conjugation and consequently a large TPABCS.^[^ ^25^ ^]^ Moreover, the decreased distortion also increased the Δ*E*_ST_ value of BNPs, synergistically inhibiting ROS generation and improving FQY.

### 2.3 Generalizability of Post‐Regulation Strategy

**Figure 3:**
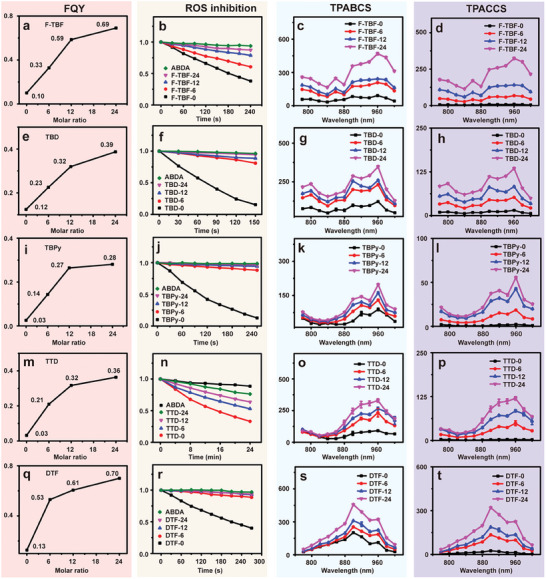
The generality of a post‐regulation strategy of AIEgens’ photophysical properties. The FQY, decomposition rate of ABDA in the absence/presence of BNPs at 399 nm under light irradiation for different times, TPABCS, and TPACCS of BNPs of a–d) TBF (encapsulated with F127), e–h) TBD, i–l) TBPy, m–p) TTD and q–t) DTF.

To investigate the generality of the proposed post‐regulation strategy, another triblock polymer, Pluronic F127, was used as the encapsulation polymer. TBF was encapsulated with Pluronic F127 by the same procedure. Similar photophysical properties, including size distribution (Figure S17, Supporting Information), blue‐shifted emission (Figure S18, Supporting Information), FQY enhancement by 6.9 times (**Figure** **3**a), ROS generation suppression (Figure 3b), and TPABCS and TPACCS enhancement by 5.1 and 35.6 times, respectively (Figure 3c,d), were obtained (Figure 3a–d; Figure S17,S18, Supporting Information) as the binary molecular ratio increased.

To further verify the generality of the post‐regulation strategy for functional AIEgens, another four typical AIE fluorescence agents (TBD, D‐A‐A structure with tetraphenylethylene as the AIE moiety, neutral molecule; TBPy, D‐A‐A structure with triphenylamine as the AIE moiety, positive molecule; TTD, D‐D‐A structure with triphenylamine as the AIE moiety, neutral molecule; and DTF, D‐A‐D structure with both triphenylamine and fumaronitrile as the AIE moieties, neutral molecule) were designed and synthesized to form BNPs (Figure S19, Supporting Information; Figure 3e–t; Figures S20–S34, Supporting Information and Experimental Section for details) by encapsulating with DSPE‐mPEG. The FQY of these BNPs‐24 was enhanced by 3.1, 11.1, 11.4, and 5.5 times (Figure 3e,i,m,q) as compared with TBD‐0, TBPy‐0, TTD‐0, and DTF‐0, respectively. Moreover, once mixed with TPE‐Br, the ROS generation capability of TBD‐24, TBPy‐24, TTD‐24, and DTF‐24 was significantly suppressed or even eliminated (Figure 3f,j,n,r). In addition, the TPABCS of these AIEgens were also significantly increased by 2.9, 2.2, 3.6 and 2.3 times (Figure 3g,k,o,s), and the TPACCS were increased by 9.0, 24.4, 41.0 and 12.7 times (Figure 3h,l,p,t) within BNPs‐24, respectively. All these experimental results demonstrated the feasibility and generality of improving the photophysical properties by the proposed post‐regulation strategy.

### 2.4 Biocompatibility and Biodistribution Investigation of TBF‐24

Before performing the intravital TPF imaging studies, the biocompatibility and biodistribution of TBF‐24 were systematically investigated. TBF‐24 with different concentrations was incubated with normal cells and cancer cells for different incubation times respectively, and the cell viability was evaluated using standard Cell Counting Kit‐8 (CCK‐8). There was no significant difference between the control cells and cells treated with TBF‐24 in the concentration range of 6.25–150 µg mL^−1^ (9.69–233 µm) for 24 and 48 h (Figure S35a,b, Supporting Information), indicating excellent in vitro biocompatibility. The in vitro biocompatibility of TBF‐24 was further investigated by hemolysis assay (Figure S35c, Supporting Information), no significant hemolysis (<5%) was observed when TBF‐24 (300 µg mL^−1^, based on TBF) was incubated with red blood cells (RBCs) for 12 h.

In addition, in vivo biocompatibility was also studied to evaluate the imaging agent. The blood was collected for routine hematology assays and blood biochemistry analysis on days 1, 8, and 15 after administration of TBF‐24 (5 mg kg^−1^, based on TBF) via subcutaneous injection. As shown in Figure S36 and Table S1 (Supporting Information), no significant abnormities in hematology parameters and biochemical indicators, related to the hepatic function and kidney function, were observed, indicating the synthesized TBF‐24 had no obvious in vivo toxicity. Meanwhile, the body weight was monitored every day within 15 days after intravenous injection of TBF‐24 (5.0 mg kg^−1^, based on TBF), and there were no obvious changes as compared with the control one (Figure S35d, Supporting Information). On day 15, the major organs including the brain, heart, liver, spleen, lung, and kidneys were collected from sacrificed mice, which were treated with TBF‐24 (5.0 mg kg^−1^, based on TBF) or PBS, for analysis via H&E staining. There were no signs of organ lesions in mice of all groups (Figure S37, Supporting Information). All these results indicate that TBF‐24 has excellent in vivo biocompatibility.

The ex vivo fluorescence images of major organs excised from mice (Female ICR mice, 5 weeks old) at 4, 8, 12, 24, 48, 72, and 96 h post intratumoral injection of TBF‐24 (5.0 mg kg^−1^) were acquired to study the biodistribution of the administrated TBF‐24 (Figure S38, Supporting Information). The liver showed the strongest fluorescence intensity among all the organs, indicating that the injected TBF‐24 was mainly accumulated in the liver. Meanwhile, the fluorescence intensity in the liver reached the maximum at 12 h post‐injection and then decreased to 38% of the maximum at 96 h post‐injection, indicating that the TBF‐24 could be efficiently cleared out via hepatic clearance.

### 2.5 Intravital TPF Imaging of Cerebrovascular Network

**Figure 4:**
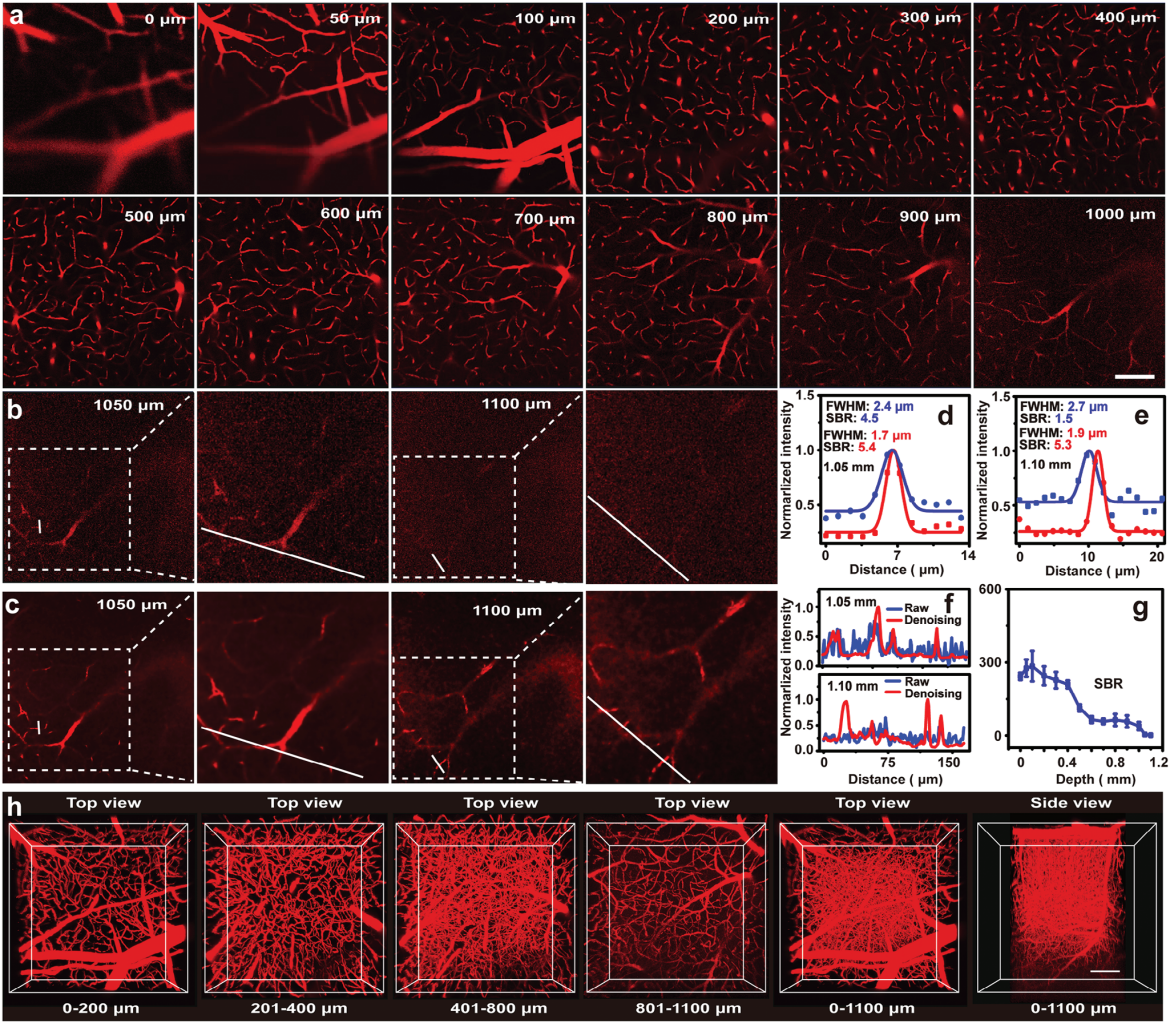
Intravital TPF images of mouse brain blood vessels. a) The raw images of mouse cerebrovascular at different vertical depths (0‐1.0 mm). The b) raw and c) denoised TPF images at vertical depths of 1.05, and 1.10 mm and the enlarged area within the dashed box. Resolution and SBR analysis of TPF imaging at depths of d) 1.05 and e) 1.10 mm before (blue) and after (red) denoising. f) The fluorescence intensity analysis along the white lines in (b) and (c) at vertical depths of 1.05 (top) and 1.10 mm (bottom), respectively. g) The SBR analysis of raw TPF images at different depths. h) 3D reconstruction images of brain vasculature. The white dotted square, short white line, and long white line on the images of (b) and (c) indicated the selected regions for enlarged areas, resolution analysis, and fluorescence intensity analysis areas, respectively. TBF‐24: 5 mg kg^−1^, Ex: 960 nm, power: 45 mW, scale bar: 100 µm.

Due to its superior biocompatibility and efficient hepatic clearance (Figures S35–S38 and Table S1, Supporting Information), TBF‐24 was suitable for intravital TPF imaging. After the skull was opened up through microsurgery (Figure S39, Supporting Information), the mouse was injected with TBF‐24 via the tail vein, which was followed with the TPF imaging (**Figure** **4**). The cerebrovascular network within 1.10 mm beneath the brain surface including big vasculatures in the shallow brain layer and tiny capillaries in the deep brain layer were clearly visualized (Figure 4a,b). Resolutions, estimated using full width at half‐maximum (FWHM), could reach 2.4 and 2.7 µm at depths of 1.05 and 1.10 mm, respectively (Figure 4d,e, blue). Moreover, the SBR could reach as high as 284 at the superficial layer (100 µm) (Figure 4g), enabling high‐contrast intravital vasculature imaging. The SBR decreased with increasing imaging depth due to the scattering. However, the SBR still reached 4.5 and 1.5 even when the imaging depth reached 1.05 and 1.10 mm (Figure 4d,e, red), respectively, ensuring excellent imaging contrast. The re‐constructed 3D images of the cerebrovascular network could clearly display the major blood vessels, capillaries, and junctions (Figure 4h). The achieved ultradeep intravital TPF imaging capability could be attributed to high FQY, large TPABCS, and FR/NIR emission of TBF‐24, which were achieved by simple post‐regulation. Meanwhile, TPF imaging parameters were summarized in Table S2 (Supporting Information) and compared with previous reports. It was worth noting that some larger imaging depth was reported, but it was achieved with special excitation parameters, such as NIR‐II excitation wavelength, and extremely high excitation power (1.2 W).^[^ ^26^ ^]^

Considering that the mouse brain was highly scattering tissue, the TPF imaging of the cerebrovascular network in the deep brain layer suffered from low SBR. To further improve the imaging quality, particularly in the deep brain layer, self‐supervised denoising algorithms were employed (Figure S40, Supporting Information). The adapted DeepCAD‐RT denoising model was trained to eliminate noise in the input images,^[^ ^27^ ^]^ and could efficiently enhance SBR and clearly reveal vascular structures without introducing visible artifacts. (Figure 4c,f; Figure S41, Supporting Information). For quantitative analysis, SBR and FWHM of the denoised TPF images were calculated and compared (Figure 4c,f). At depths of 1.05 and 1.10 mm, SBR increased from 4.5 and 1.5 to 5.4 and 5.3, respectively, and the FWHM was reduced from 2.4 and 2.7 to 1.7 and 1.9 µm (Figure 4d,e), respectively. Moreover, some details that are unrecognizable in the original noisy region become visible (Figure 4b,c,f). The adapted DeepCAD‐RT network could significantly improve the SBR and resolution of TPF imaging particularly in the deep brain layer.

Inspired by the high performance of TBF‐24 in intravital TPF imaging, we further explored cerebrovascular network imaging through intact skulls. The TPF images clearly revealed the blood vessels with high resolution and good SBR at various depths beneath the skull (Figure S42, Supporting Information), the maximal imaging depth reached 400 µm, which was better than other reported results (≈300 µm)^[^ ^28^ ^]^ and comparable to that of excitation at NIR‐II window (≈400 µm).^[^ ^29^ ^]^ These results demonstrated that TBF‐24 based TPF imaging through intact mouse skulls was achievable, but it was limited by strong optical scattering in turbid brain tissue and skull. Therefore, a thinned skull window was prepared for intravital TPF imaging. The cerebrovascular network, including arteries, capillaries, and veins, was clearly distinguished within 750 µm beneath the surface (Figure S43, Supporting Information). The resolution and SBR could reach 1.8 µm and 3.3 at the depth of 500 µm, respectively. Thus, the TPF imaging through a thinned skull window possessed not only high resolution and contrast but also large imaging depth, which were particularly important for long‐term intravital studies of vascular events.

### 2.6 Intravital TPF Imaging of Metastatic Cancer Cells with Single‐Cell Resolution

**Figure 5:**
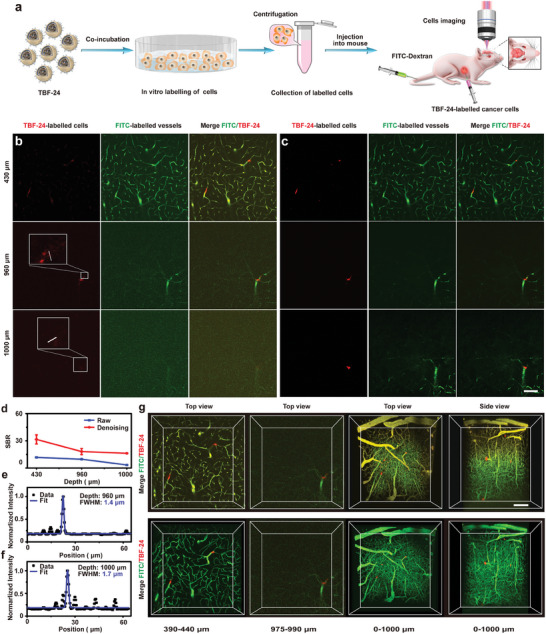
Intravital TPF imaging of metastatic cancer cells in mouse brain. a) Scheme of two‐channel TPF imaging of metastatic cancer cells in the cerebrovascular network. Representative b) raw, c) denoised and color unmixed TPF images of the TBF‐24‐labelled 143B cells (red channel) and the FITC‐Dex‐labelled vessels (green channel), and merged images at vertical depths of 430, 960, 1000 µm. The dashed region shows the enlarged image. d) The SBR analysis of the raw (blue) and denoised (red) TPF imaging (red channel) at different depths. Resolution analysis of the raw TPF imaging (red channel) at depths of e) 0.96 and f) 1.0 mm. The white line on the image indicates the selected region for resolution analysis. g) The raw (top) and color unmixing (bottom) 3D reconstruction of the TBF‐24‐labelled 143B cells (red channel) and FITC‐Dex‐labelled brain vasculature (green channel), and merged images. TBF‐24: 5 mg kg^−1^ (based on TBF), FITC‐Dex: 240 mg kg^−1^, Ex: 960 nm, power: 45 mW. Scale bars, 50 µm.

Intravital TPF imaging is an important tool to observe cellular behavior in live animals. The TBF‐24‐based TPF imaging has achieved high‐performance imaging of the cerebrovascular network, thus it was applied for real‐time intravital imaging of metastatic cancer cells, which has been recognized as a key factor in inducing the high death rate during cancer development and prognosis.^[^ ^30^ ^]^ TBF‐24‐labelled human osteosarcoma 143B cells were intracardially injected to simulate brain metastases (**Figure** **5**a). Meanwhile, an ultra‐high dose of FITC‐Dex was injected via the tail vein to stain blood vessels and consequently map the deep cerebrovascular network and create a reference for the metastatic cancer cells, which was followed by intravital TPF imaging for analysis (Figure 5b–g). The two‐channel TPF images revealed that cancer cells were arrested in vessel bifurcations through physical occlusion (Figure 5b), which was in good agreement with the previously reported cancer metastasis mechanism.^[^ ^31^ ^]^ Moreover, the SBR of metastatic cancer cell images could reach 9.7 and 3.4 even at the depths of 0.96 and 1.0 mm, respectively (Figure 5d), and the resolution of accumulated cells was 1.4 and 1.7 µm at the depths of 0.96 and 1.0 mm (Figure 5e,f). It could be deduced that intravital TPF imaging could achieve single‐cell resolution,^[^ ^32^ ^]^ which was unachievable for common TPF probes at such a deep cerebrovascular network. The 3D reconstruction images of the TBF‐24‐labelled 143B cells (red channel) and FITC‐Dex‐labelled cerebrovascular network (green channel) clearly displayed the vessels, cells, and their spatial distribution information, demonstrating cells were arrested through physical occlusion in vessel bifurcation (Figure 5g).

The DeepCAD‐RT denoising model was also trained to improve the imaging quality of metastatic cancer cells and cerebrovascular network. The signals, particularly the weak signals, were successfully resolved and accurately mapped (Figure 5c), and even some fine structures indistinguishable in the raw images could be recognized after denoising. The image quality was quantified by FWHM and SBR analysis (Figure 5e,f), the denoised images eliminated noise‐induced resolution degradation, improving SBR at the depth of 0.96 and 1.0 mm from 9.7 and 3.4 to 18.2 and 16.3, respectively.

In addition, spectral bleed‐through is a common phenomenon in dual and multi‐channel imaging due to the spectral overlap of fluorescent chromophores and the non‐exclusive features of optical filters in the corresponding channel. The yellowish signal appeared in the merged images, which was derived from the spectral bleed‐through of red and green channels. A color unmixing algorithm was used to separate the two channels to reduce the degradation of merged images, thereby maintaining high accuracy and fidelity (Figure 5c,g).

## 3 Discussion

TPF imaging is a critical tool to visualize cellular behavior, tissular morphology, and biological processes due to its distinguished advantages including high sensitivity, excellent spatial and temporal resolution, etc. However, the currently available TPF agents of AIEgens suffer from small TPABCS, low FQY, and subsequently small TPACCS, leading to extremely high‐power excitation and low SBR. To regulate and optimize the photophysical properties, iterative design and synthesis are inevitable, which may introduce undesired phototoxicity. Furthermore, the optimization strategy has no generality. Here, we proposed a general and efficient post‐regulation strategy to improve the photophysical properties of AIEgens, which simultaneously endowed the TBF‐24 with 8.7‐fold enhanced FQY, 5.4‐fold enhanced TPABCS, negligible phototoxicity, etc. The generality of the proposed post‐regulation strategy was also demonstrated using another of our typical functional AIEgens and encapsulation matrices. The proposed post‐regulation strategy significantly improved the intravital TPF imaging of cerebrovascular network and metastatic cancer cells, endowing large imaging depth of ≈1.10 mm and high imaging resolution of single‐cell resolution.

The post‐regulation capability can be attributed to (I), the polar TBF was separated by the nonpolar TPE‐Br, leading to an increased FQY due to a weaker dipole‐dipole interaction. The decreased polarity and dipole‐dipole interaction were confirmed by fluorescence lifetime measurement, (II) the increased Δ*E*_ST_ of TBF, which was calculated by HOMO and LUMO distribution simulation and indicated by the spectral response, significantly inhibited the energy dissipation pathways of ISC, enabling the absorbed excitation energy mainly released by fluorescence emission. Remarkably decreased ROS generation and enhanced FQY were achieved, making BNPs a promising TPF imaging agent with negligible phototoxicity, (III) the decreased distortion angles, calculated by dihedral angles simulation, induced intramolecular D‐A conformation change from twisted to coplanar structure, which was in favor of yielding better conjugation and consequently a large TPABCS. The post‐regulation mechanism was investigated by both experimental measurement and theoretical simulation, which matched well with each other. These improvements enabled high‐quality TPF imaging of cerebrovascular networks using BNPs with a large imaging depth of 1.10 mm and high resolution of 2.7 µm (at the depth of 1.10 mm), the high resolution enabled the detection of metastatic cancer cells with single‐cell resolution. The imaging quality could be further improved by a self‐supervised denoising algorithm.

The proposed method is versatile and easy to operate by simply tuning the ratio of two types of synthesized or commercial AIEgens, making it a fruitful path for developing high‐performance TPF agents using existing AIEgens. Meanwhile, it is intuitive to operate and readily adopted by researchers who have no training in organic synthesis. Considering its generality across a series of typical functional AIEgens and encapsulation matrices, the post‐regulation strategy could be a promising method for elevating the performance and expanding the application scope of TPF imaging.

## 4 Experimental Section

### Synthesis and Characterization

See the Supplementary Materials for the detailed synthesis characterization information.

### Preparation of BNPs

TBF and TPE‐Br were dissolved in THF. To obtain BNPs with different ratios of TBF to TPE‐Br, 100 µL TBF solutions (1.5 mm) were added to 0, 100, 200, 400, and 600 µL of TPE‐Br solution (9 mm), respectively. Then, THF was added to each of the five solutions until the volume reached 2 mL. DSPE‐mPEG, which was twice the total mass of TBF and TPE‐Br, was subsequently added to each mixture solution. Finally, each of the mixture solutions was injected into 18 mL of deionized water and sonicated for 2 min. BNPs aqueous solutions were obtained after THF was volatilized by stirring at room temperature for 12 h. The BNPs of TBD, TBPy, DTF, and TTD were prepared using similar procedures. The mixture solutions were then concentrated by ultrafiltration (30 K Amicon Ultra filter, Millipore Corporation). Finally, the BNPs solutions were obtained and stored at 4 °C. Before intravenous injection, potential aggregates were removed by filtration using a 0.22 µm filter.

### Fluorescence Quantum Yield Measurement

The FQY (*η*) was measured using Rhodamine 6G as the reference (*η* = 0.95 in ethanol), and the relative fluorescence quantum yield was calculated according to the equation:^[^ ^33^ ^]^

(1) $$\eta_{x}=\left(\frac{A_{s}}{A_{x}}\right)\left(\frac{F_{x}}{F_{s}}\right)\left(\frac{n_{x}^{2}}{n_{s}^{2}}\right)\eta_{s}$$

where *η* is the FQY, *A* is the absorbance spectra intensity, and *F* is the fluorescence spectra integrated area. *n* is the refractive index of the solvents. The subscripts s and x refer to the standard and the sample, respectively.

### The Two‐Photon Absorption Cross‐Section Measurement

The TPABCS was measured using a two‐photon‐induced fluorescence method.^[^ ^34^ ^]^ A home‐built microscopy equipped with tunable femtosecond (fs) laser (Coherent, Chameleon Ultra II) was applied (Figure S13, Supporting Information), the sample solutions were deposited on the confocal 96 wells, TPF signals were collected by an objective (10×, NA = 0.3) under xyλ. Rhodamine 6G in methanol was used as the reference. The TPABCS can be calculated according to the equation:

(2) $$\sigma_{x}=\left(\frac{F_{x}}{F_{s}}\right)\left(\frac{\eta_{s}}{\eta_{x}}\right)\left(\frac{c_{s}}{c_{x}}\right)\left(\frac{n_{s}}{n_{x}}\right)\sigma_{s}$$

where *F* is the TPF intensity, *η* is the fluorescence quantum yield, *c* is the molar concentration, and *n* is the refractive index of the solvents. The subscripts s and x refer to the standard and the sample, respectively.

### Singlet Oxygen Detection

The ^1^O_2_ generation efficiencies were assessed by ABDA, a ^1^O_2_ indicator. In the presence of ^1^O_2_, the absorbance of ABDA would significantly decrease due to the oxidative decomposition effect. Briefly, a 2 mL solution containing 50 µM ABDA and 6.5 µM NPs was exposed to light irradiation (400–700 nm, 70 mW cm^−2^) for different times, then the corresponding absorption spectra were recorded immediately.

### Photostability Evaluation

The anti‐photobleaching capability of TBF‐24 was evaluated by recording the TPF signal under continuous excitation and comparing it with that of one commercial fluorophore, i.e., Evans blue. Briefly, the same final concentrations (10 µM) of TBF‐24 and Evans blue aqueous solution were added to the confocal dish, respectively, which was followed by continuous irradiation for different times with femtosecond laser (960 nm, 30 mW). Finally, the TPF intensity of TBF‐24 and Evans blue was recorded and compared.

### Cellular Internalization and TPF Imaging

HeLa, 143B, and HEK293 cells were seeded on confocal dishes and cultured overnight to ≈80% coverage. TBF‐24 (50 µM based on TBF) were incubated with cells for 4 h. After washing three times with PBS to remove the residual TBF‐24, the TBF‐24‐stained cells were processed for TPF imaging. The TPF imaging was exciting with a femtosecond laser (960 nm, 30 mW), and the one‐photon fluorescence (OPF) imaging was excited at 488 nm. The TPF and OPF were corrected by 20 × objective lenses (Olympus, XLUMPLFLN20XW).

### Cytotoxicity Assay

Cellular viability was assessed by the CCK‐8 assay. The cells were planted on 96‐well plates with a density of 5000 cells per well and incubated for 12 h. The culture medium containing various concentrations of TBF‐24 was added to the well to replace the culture medium. After incubation for 24 and 48 h, 90 µL of fresh cell culture medium and 10 µL of CCK‐8 were added into each well and incubated for 2 h. To eliminate the influence of weak absorption from BNPs‐24 at 450 nm, the cells incubated with various concentrations of TBF‐24 were defined as the baseline. Finally, the absorbance at 450 nm was measured using a microplate reader. The cell viability was calculated using the following equation:

(3) $$Cell\;viability\;\left(\%\right)=\left(OD-OD_{Baseline}\right)/\left(OD_{Control}-OD_{Blank}\right)$$

### Evaluation of Hemolysis

The blood samples were collected from the BALB/c mice and centrifuged at 5000 rpm for 5 min and washed five times with PBS. 2% erythrocytes (v/v) containing various concentrations of TBF‐24 solution (25, 50, 100, 200, 300 µg mL^−1^), water and saline were incubated for 12 h at 37 C. The mixture solutions were centrifuged at 3000 rpm for 6 min and the supernatants were collected. The released hemoglobin in the supernatant was analyzed by recording the absorbance at 542 nm by a microplate reader. The percentage of hemolysis was calculated as follows:

(4) $$Hemolysis\;\left(\%\right)=\left(I_{sample}-I_{B}-I_{0}\right)/\left(I_{100}-I_{0}\right)\times 100$$

where *I*_sample_, *I*_B_, *I*_100,_ and *I*_0_ referred to the absorbance of the erythrocytes with different concentrations of TBF‐24, the TBF‐24 solution, the completely lysed red blood cells in distilled water, and no obvious hemolysis in PBS. All hemolysis assays were conducted with three replicates (n = 3).

### Animals

All animal experiments were approved by the Ethical Committee of Animal Experiments in the School of Biomedical Engineering, Shanghai Jiao Tong University (A2023031), and were consistent with regulations for the care and use of experimental animals in China. All animals were group housed in a standard 12 h light/12 h dark cycle with ad libitum access to food and water. All mice were purchased from the Shanghai Laboratory Animal Center.

### In Vivo Systemic Biotoxicity Study

Female ICR (Institute of Cancer Research) mice (5 weeks old) were used to evaluate the biotoxicity, the mice were divided into two groups. Control group: intravenous injection of PBS (50 µL); TBF‐24 group: intravenous injection of TBF‐24 (5 mg kg^−1^, 50 µL). The blood was collected on days 1, 8, and 15. The hepatic function and kidney function parameters, including alkaline phosphatase (ALP), aspartate aminotransferase (AST), alanine transaminase (ALT), creatinine (CREA), and blood urea nitrogen (BUN), were monitored. For the blood routine analysis, indexes such as red blood cells (RBC), hematocrit (HCT), hemoglobin (HGB), platelets (PLT), mean platelet volume (MPV), mean corpuscular volume (MCV) and mean corpuscular hemoglobin concentration (MCHC) were evaluated.

### Animals for Intravital TPF Imaging of Brain Blood Vessels

Female ICR mice (7 weeks old) were anesthetized, and the scalp was further sterilized with alcohol wipes, and then a section of the scalp was removed to expose the skull. Fascia and connective tissue on the skull were gently removed with forceps and sterile wet cotton tips to avoid any internal bleeding inside the brain. A small piece of the mouse skull was removed to allow the transmission of light, and a coverslip (5 mm diameter, 0.15 mm thickness) was pressed closely and then glued. Dental cement was applied to the edges of the coverslip, and a slight berm was created using cement to hold water for dipping the objective lens. Finally, the mouse was fixed on a custom‐made stereotaxic plate, and then placed on a heat blanket to maintain body temperature at 37.5 °C. 50 µL of TBF‐24 (5 mg kg^−1^, based on TBF) was administered via intravenous for TPF imaging. The intravital TPF imaging was performed immediately after the administration of TBF‐24. The TPF images of brain blood vessels were captured with a 2 µm step (2 µs/pixel, 512 × 512 pixels).

### Intravital TPF Imaging of Metastatic Cancer Cells in Mouse Brain

Human osteosarcoma 143B cells were incubated with TBF‐24 (200 µM) for 4 h. After washed three times with PBS, the labeled 143B cells were digested, centrifuged, and resuspended to obtain cell suspension. For the intracardiac injection, 100 µL PBS (1×) containing 1 × 10^6^ TBF‐24‐labelled 143B cells was injected into the left ventricle. To visualize the cerebral vessels, 100 µL PBS (1×) containing 60 mg mL^−1^ FITC‐dextran (150 kDa) was injected through the tail vein. Finally, the ICR mouse was placed on a stereoscopic locator with the head fixed for TPF imaging. The intravital TPF imaging was immediately performed after administration.

### Neural Network Architecture

The adapted DeepCAD‐RT denoising model was trained as a self‐supervised denoising learning method to improve imaging quality. The model was trained in a generative‐adversarial fashion. The generator was based on a 3D U‐Net in the encoder‐decoder architecture, with four down‐sampling stages and four up‐sampling stages. The training data consisted of five 3D stacks with the size of 300*512*512 pixels in numpy array format. The input training 3D stacks were divided into smaller sub‐stacks (120*150*150) as training patches, with a lateral overlap region of 90 pixels (overlap_factor = 0.6), since the overlapping region was required to be larger than 88 pixels, the size of the reception field of 3D U‐Net. Adjacent patches were used as input and target image pairs. The network was trained for 15 epochs, with a learning rate of 0.0001.

In the deployment stage, before the 3D stack (300*512*512) was processed by the self‐supervised denoising model, the mean value of the stack was subtracted to handle the variation of the stack. Then, the 3D stack was divided into sub‐stacks of 90*150*150, with a lateral overlap region of 90 pixels (overlap_factor = 0.6). After denoising, the mean value was added back to the stack to yield the final denoised stack.

The graphics hardware used for training and deployment is NVIDIA GeForce RTX 3050 Ti Laptop GPU. The processing unit is Intel Core i7‐12700H (2.30 GHz), with a RAM of 16 GB.

### Color Unmixing

To eliminate the spectral bleed‐through in the merged images, a customized color unmixing algorithm was adapted and applied. The color unmixing algorithm retained the red channel signal in image areas where the red channel signal is higher than the green channel signal by a threshold T (T = 80) and set the red channel signal to zero in image areas where the difference of red channel signal and green channel signal was lower than the threshold T. After thresholding, a gamma correction step was applied to the image, where gamma was set to 0.16. Finally, additional gamma adjustment was applied using exposure, adjust, and sigmoid function of the image library.

### Statistical Analyses

All data points or bars are shown as the mean ± standard deviation (SD) from at least three independent experiments. All statistical evaluation was performed with GraphPad Prism 8.0 software (Version 10.2.3), and data were analyzed by one‐way analysis of univariate variance (ANOVA) or unpaired Student's t‐test. For all numerical tests, a probable value (**, p < 0.01, ***, p < 0.001, ****, p < 0.0001, ns: not significant) was considered to be statistically significant.

## Conflict of Interest

The authors declare no conflict of interest.

## Author Contributions

L.L and J.L contributed equally to this work. B. Gu and L. Lin conceived the idea. B. Gu, L. Lin, and W. Wu designed the research. L. Lin, J. Liu, W. Pang, X. Jiang, M. Lei, J. Gao, and Y. Xiao performed the experiments. Z. Pan performed the self‐supervised denoising algorithm. L. Lin, J. Liu, Z. Pan, B. Li, F. Hu, Z. Bao, X. Wei, W. Wu, and B. Gu analyzed the data. B. Gu, L. Lin, B. Li, and W. Wu wrote and revised the manuscript, and all authors reviewed the manuscript.

## Supporting information

Supporting Information

## Data Availability

The data that support the findings of this study are available from the corresponding author upon reasonable request.
