# Supplementary material for: General Post‐Regulation Strategy of AIEgens’ Photophysical Properties for Intravital Two‐Photon Fluorescence Imaging
Source: Adv Sci (Weinh). 2024 Aug 9;11(38):2404792. doi: 10.1002/advs.202404792 (PMC11481373; doi:10.1002/advs.202404792)
Supplement: Supplementary file 1 — Supporting Information [file ADVS-11-2404792-s001.pdf]

ADVANCED SCIENCE
Open Access

Supporting Information

for *Adv. Sci.*, DOI 10.1002/advs.202404792

General Post-Regulation Strategy of AIEgens' Photophysical Properties for Intravital Two-Photon Fluorescence Imaging

Liyun Lin, Jiaxin Liu, Zhengyuan Pan, Wen Pang, Xinyan Jiang, Man Lei, Jucai Gao, Yujie Xiao, Bo Li, Fang Hu, Zhouzhou Bao, Xunbin Wei*, Wenbo Wu* and Bobo Gu**

Supporting Information

General Post-regulation Strategy of AIEgens' Photophysical Properties for Intravital Two-photon Fluorescence Imaging

Liyun Lin[1,†], Jiaxin Liu[2,†], Zhengyuan Pan[1], Wen Pang[1], Xinyan Jiang[1], Man Lei[1], Jucai Gao[3], Yujie Xiao[4], Bo Li[4], Fang Hu[3], Zhouzhou Bao[5, *], Xunbin Wei[6,*], Wenbo Wu[2,*], Bobo Gu[1,*]

[1]School of Biomedical Engineering, Shanghai Jiao Tong University, Shanghai 200030, China

[2]Department of Chemistry, Institute of Molecular Aggregation Science, Tianjin University, Tianjin 300072, China

[3]Biomaterials Research Center, School of Biomedical Engineering, Southern Medical University, Guangzhou 510515, China.

[4]Department of Neurology, Huashan Hospital, MOE Frontiers Center for Brain Science, State Key Laboratory of Medical Neurobiology, Institutes for Translational Brain Research, Fudan University, Shanghai 200437, China

[5]Shanghai Key Laboratory of Gynecologic Oncology, Ren Ji Hospital, School of Medicine, Shanghai Jiao Tong University, Shanghai 200127, China

[6]Biomedical Engineering Department and International Cancer Institute, Peking University, Beijing 100191, China

[†]These authors contributed equally to this work.

*Corresponding author. Email: baozhouzhou@126.com (Zhouzhou Bao), xwei@bjmu.edu.cn (Xunbin Wei), wuwb@tju.edu.cn (Wenbo Wu), bobogu@sjtu.edu.cn (Bobo Gu)

Experimental methods and materials

Materials

Ultra-dry dichloromethane, toluene, dioxane, *N*, *N*-dimethylformamide (DMF), and tetrahydrofuran (THF) were obtained through a commercial solvent purification system. Anhydrous ethyl ether was provided as chromatographic purity. TBD and DTF were prepared according to our previous work[1], while TTD was prepared according to the procedure reported in the literature[2]. All other reagents were used as received unless otherwise specified. THF, Rhodamine 6G, dimethyl sulfoxide (DMSO), Pluronic F127 (F127) and 9,10-Anthracenediyl-bis(methylene)dimalonic acid (ABDA) were purchased from Sigma. 1,2-distearoyl-sn-glycero-3-phosphoethanolamine-N-[methoxy(polyethylene glycol)-2000] (DSPE-mPEG) was purchased from Laysan. Cell Counting Kit-8 (CCK-8) was purchased from Dojindo. 1,1,2,2-Tetrakis(4-bromophenyl)ethylene (TPE-Br) was purchased from Macklin Biochemical. Evans blue was purchased from Shanghai Yuanye Bio-Technology. HeLa (CCL-2), 4T1 (CRL-3404), HEK293 (CRL-1573), and 143B (CRL-8303) cells were purchased from ATCC. DMEM medium and phosphate-buffered saline (PBS) were purchased from HyClone. Fetal calf serum was purchased from ScienCell. Antibiotics (penicillin and streptomycin mixture) were purchased from Gibco. FITC-dextran (FITC-Dex, 150 kDa) was purchased from QIYUEBIO.

Instruments

^{1}H, ^{13}C, and ^{19}F NMR spectra were measured by Bruker Avance III spectrometer (400 MHz) using tetramethylsilane (TMS; δ = 0 ppm) as the internal standard. The mass spectra were recorded by matrix-assisted laser desorption ionization (MALDI) UltrafleXtreme time of flight (TOF) equipment (Bruker Daltonics, USA). HR-ESI-MS data were obtained with a Q Exactive HF Orbitrap LC-MS (Thermo Fisher Scientific, USA). The UV-vis absorption spectra were measured using a UV1901PC spectrophotometer (Aucy, China). Dynamic light scattering (DLS) was measured by Zetasizer Nano ZSP (Malvern, UK). The fluorescence and phosphorescence spectra were measured by an FLS1000 spectrofluorometer (Edinburgh Instruments, UK). The two-photon fluorescence spectra and cellular imaging were measured by laser confocal scanning microscopy (CLSM) (Leica SP8, Germany) equipped with a tunable femtosecond laser (Coherent, Chameleon Ultra II, 700-1040 nm, 130 fs, 3.8 W, 80 MHz). The absorbance of CCK-8 at 450 nm and hemoglobin at 542 nm were recorded using SpectraMax M5 Microplate Reader (Molecular Devices, USA). Upright microscopy (Olympus, FVMPE-RS; objective: XLUMPLFLN20XW) equipped with a tunable fs laser was applied for intravital TPF imaging.

Synthesis of 2,3-bis(4-bromophenyl)fumaronitrile (2)

A solution of 4-bromophenylacetonitrile (1) (3.92 g, 20.00 mmol), iodine (5.08 g, 20.00 mmol) and anhydrous ethyl ether (70 mL) was cooled to -78 °C, then another cooled (-78 °C) solution of sodium methoxide (25% solution in methanol) (9.83 mL, 43.00 mmol) in methanol (15 mL) was added to the reaction system and stirred for 30 min at -78 °C. Next, the resultant mixture was placed in an ice bath and stirred at 0 °C for further 4 h. Afterwards, the resultant mixture was quenched by the addition of HCl (aq) (3%, v/v, 60 mL) and then the precipitates were filtered and washed three times in the order of water (100 mL), $Na_2S_2O_5$ (aq) (5%, v/v, 50 mL) and water (100 mL). Finally, pure product 2 was obtained by recrystallization from ethanol as a pale-yellow solid (2.82 g, 73%). ^{1}H NMR (400 MHz, $CDCl_3$, 298K), δ (TMS, ppm): 7.73-7.65 (m, 8H).

Synthesis of 2-(4-bromophenyl)-3-(4'-(diphenylamino)-[1,1'-biphenyl]-4-yl)fumaronitrile (4)

Compound 2 (776.1 mg, 2.00 mmol), (4-(diphenylamino)phenyl)boronic acid (3) (289.1 mg, 1.00 mmol), potassium carbonate (1.38 g, 10.00 mmol), and $Pd(PPh_3)_4$ (3%) were placed into a 200 mL Schlenk tube, degassed and charged with nitrogen for three times. Then the mixture was dissolved in THF (60 mL)/water (10 mL) under nitrogen and stirred at 60 °C for 12 h. After the reaction system was brought to room temperature, a large amount of water was added and extracted with dichloromethane. The organic layer was dried over anhydrous sodium sulfate and evaporated to remove the solvent. Then the crude product was purified by column chromatography on silica gel by using petroleum ether/dichloromethane (4/1, v/v) as the eluent to afford an orange solid 4 (333 mg, 60%). ^{1}H NMR (400 MHz, $CDCl_3$, 298K), δ (TMS, ppm): 7.91 (d, J = 8.4 Hz, 2H, ArH), 7.75-7.64 (m, 6H, ArH), 7.51 (d, J = 8.6 Hz, 2H, ArH), 7.32-7.26 (m, 4H, ArH), 7.17-7.11 (m, 6H, ArH), 7.07 (t, J = 7.4 Hz, 2H, ArH). ^{13}C NMR (100 MHz, $CDCl_3$, 298K), δ (ppm): 148.52, 147.40, 144.38, 132.69, 132.44, 131.21, 130.30, 129.84, 129.52, 129.34, 127.95, 127.17, 126.42, 125.85, 125.01, 123.61, 123.18, 122.79, 116.81, 116.62.

Synthesis of phenyl(4-(4,4,5,5-tetramethyl-1,3,2-dioxaborolan-2-yl)phenyl)methanone (5)

4-Bromobenzophenone (1.30 g, 5.0 mmol), bis(pinacolato)diborane (2.54 g, 10.0 mmol), potassium acetate (1.72 g,

17.5 mmol), Pd(dppf)Cl_2 (5%) were placed into a 100 mL Schlenk tube, degassed and charged with nitrogen for three times. The mixture was dissolved in dioxane (20 mL) under nitrogen and stirred at 85 °C for 48 h and then cooled to room temperature. A large amount of water was added and extracted with dichloromethane. The organic layer was dried over anhydrous sodium sulfate and evaporated to remove the solvent. The crude product was purified by column chromatography on silica gel by using petroleum ether/ethyl acetate (20/1, v/v) as the eluent to afford a white solid 5 (1.39 g, 90%). ^{1}H NMR (400 MHz, $CDCl_3$, 298 K), δ (TMS, ppm): 7.94-7.88 (m, 2H, ArH), 7.82-7.73 (m, 4H, ArH), 7.62-7.54 (m, 1H, ArH), 7.51-7.42 (m, 2H, ArH), 1.36 (s, 12H, -CH_3).

Synthesis of 2-(4'-benzoyl-[1,1'-biphenyl]-4-yl)-3-(4'-(diphenylamino)-[1,1'-biphenyl]-4-yl)fumaronitrile (TBF)
Compound 4 (331.5 mg, 0.60 mmol), compound 5 (308.2 mg, 1.00 mmol), potassium carbonate (829.3 mg, 6.0 mmol), and Pd(PPh_3)$_4$ (3%) were placed into a 50 mL Schlenk tube, degassed and charged with nitrogen for three times. Then the mixture was dissolved in THF (9 mL)/water (3 mL) under nitrogen and stirred at 60 °C overnight. After the reaction system was brought to room temperature, a large amount of water was added and extracted with dichloromethane. The organic layer was dried over anhydrous sodium sulfate and evaporated to remove the solvent. Then the crude product was purified by column chromatography on silica gel by using petroleum ether/dichloromethane (3/1 ~ 1/1, v/v) as the eluent to afford a red solid TBF (154 mg, 47%). ^{1}H NMR (400 MHz, $CDCl_3$, 298K), δ (TMS, ppm): 8.08-7.90 (m, 6H, ArH), 7.87-7.79 (m, 4H, ArH), 7.79-7.71 (m, 4H, ArH), 7.65-7.58 (m, 1H, ArH), 7.53 (d, J = 7.4 Hz, 4H, ArH), 7.29 (t, J = 7.8 Hz, 4H, ArH), 7.15 (d, J = 7.6 Hz, 6H, ArH), 7.07 (t, J = 7.4 Hz, 2H). ^{13}C NMR (100 MHz, $CDCl_3$, 298K), δ (ppm): 196.25, 148.49, 147.42, 144.24, 144.22, 143.40, 143.17, 137.60, 137.58, 137.29, 132.70, 132.68, 132.53, 132.02, 130.99, 130.97, 130.16, 130.13, 129.55, 129.53, 129.51, 129.38, 128.51, 128.48, 128.13, 128.11, 127.97, 127.94, 127.22, 127.14, 125.32, 125.00, 123.60, 123.58, 123.51, 117.09, 116.93. MALDI-TOF MS, calcd for ($C_{47}H_{31}N_3O$): m/z $[M]^+$: 653.246; found, m/z 653.235.

Synthesis of 4-(7-bromobenzo[c][1,2,5]thiadiazol-4-yl)-*N,N*-diphenylaniline (8)
N,N-diphenyl-4-(4,4,5,5-tetramethyl-1,3,2-dioxaborolan-2-yl)aniline (6) (0.40 g, 1.08 mmol), 4,7-dibromobenzo[c][1,2,5]thiadiazole (7) (0.48 g, 1.6 mmol) and Pd(PPh_3)$_4$ (3%) were mixed and dissolved in THF (3 mL), methanol (3 mL), and toluene (6 mL), and then stirred under argon atmosphere. Aqueous solution of K_2CO_3 (1 mL, 2 M) was then injected. The mixture continued to stir and heat at 80 °C for 23 h. After cooling to room temperature, the reaction was quenched with water, and extracted with ethyl acetate, then washed with water (20 mL × 3). The organic phase was dried with Na_2SO_4 and the solvent was removed under reduced pressure. The obtained residue was purified with chromatography (petroleum ether/dichloromethane = 15/1, v/v) to give compound 8 as a yellow solid (0.36 g, 85%). ^{1}H NMR (400 MHz, $CDCl_3$, 298K), δ (TMS, ppm): 7.89 (d, J = 7.6 Hz, 1H), 7.79 (d, J = 8.8 Hz, 2H), 7.53 (d, J = 7.6 Hz, 1H), 7.31-7.26 (m, 4H), 7.17 (dd, J = 8.6, 3.2 Hz, 6H), 7.07 (t, J = 7.2 Hz, 2H).

Synthesis of *N,N*-diphenyl-4-(7-(pyridin-4-yl)benzo[c][1,2,5]thiadiazol-4-yl) aniline (10)
Pyridin-4-ylboronic acid (9) (0.25 g, 2.0 mmol), compound 8 (0.30 g, 0.66 mmol), and Pd(PPh_3)$_4$ (3%) were dissolved in THF (10 mL), stirred under argon atmosphere. Aqueous solution of K_2CO_3 (1 mL, 2 M) was then injected. The mixture continued to stir and heat at 70 °C for 22 h. After cooling to room temperature, the reaction was quenched with water, and extracted with ethyl acetate, then washed with water (20 mL × 3). The organic phase was dried with Na_2SO_4 and the solvent was removed under reduced pressure. The obtained residue was purified with chromatography (petroleum ether/ethyl acetate = 20/1, v/v) to give compound 10 as a red solid (0.15 g, 50%). ^{1}H NMR (400 MHz, $CDCl_3$, 298K), δ (TMS, ppm): 8.77 (d, J = 6.0 Hz, 2H), 7.93 (d, J = 6.4 Hz, 2H), 7.88 (d, J = 8.8 Hz, 3H), 7.78 (d, J = 7.4 Hz, 1H), 7.30 (t, J = 7.8 Hz, 4H), 7.20 (t, J = 8.0 Hz, 6H), 7.07 (t, J = 7.3 Hz, 2H).

Synthesis of 4-(7-(4-(diphenylamino)phenyl)benzo[c][1,2,5]thiadiazol-4-yl)-1- ethylpyridin-1-ium (12)
Compound 10 (0.14 g, 0.31 mmol) and bromoethane (11) (0.34 g, 3.1 mmol) were mixed and dissolved in dry DMF (5 mL) and stirred under an argon atmosphere. The mixture continued to stir and heat at 75 °C for 48 h. After cooling to room temperature, the reaction was quenched with water, and extracted with ethyl acetate, then washed with water (20 mL × 8). The organic phase was dried with Na_2SO_4 and the solvent was removed under reduced pressure. The obtained residue was purified with chromatography (dichloromethane/methanol = 20/1, v/v) to give 12 as a red solid (148 mg, 81%). ^{1}H NMR (400 MHz, $CDCl_3$, 298K), δ (TMS, ppm): 9.54 (d, J = 6.4 Hz, 2H), 8.88 (d, J = 6.4 Hz, 2H), 8.25 (d, J = 7.4 Hz, 1H), 7.85 (dd, J = 17.2, 8.0 Hz, 3H), 7.30 (t, J = 7.6 Hz, 4H), 7.17 (d, J = 8.4 Hz, 5H), 7.15 (s, 1H), 7.12-7.07 (m, 2H), 5.08 (d, J = 7.6 Hz, 2H), 1.77 (t, J = 7.2 Hz, 3H).

Synthesis of 4-(7-(4-(diphenylamino)phenyl)benzo[c][1,2,5]thiadiazol-4-yl)-1- ethylpyridin-1-ium (TBPy)
Compound 12 (30 mg, 0.05 mmol) and potassium hexafluorophosphate (100 mg, 0.54 mmol) were mixed and

dissolved in acetone (5 mL). The mixture continued to stir and heat at 40 °C for 12 h. After cooling to room temperature, the reaction was quenched with water and ethyl acetate, and then washed with water (20 mL × 3). The organic phase was dried with Na_2SO_4 and the solvent was removed under reduced pressure. The obtained residue was purified with chromatography (dichloromethane/methanol = 20/1, v/v) to give TBPy as a red solid (25 mg, 75%). ^{1}H NMR (400 MHz, $CDCl_3$, 298K), δ (TMS, ppm): 8.79 (d, J = 7.0 Hz, 2H), 8.74 (d, J = 7.2 Hz, 2H), 8.19 (d, J = 7.6 Hz, 1H), 7.89-7.85 (m, 2H), 7.81 (d, J = 7.6 Hz, 1H), 7.33-7.28 (m, 4H), 7.20-7.15 (m, 6H), 7.12-7.08 (m, 2H), 4.68 (q, J = 7.4 Hz, 2H), 1.71 (t, J = 7.4 Hz, 3H). ^{13}C NMR (100 MHz, $CDCl_3$, 298K), δ (ppm): 153.97, 153.05, 149.47, 147.04, 143.42, 138.47, 132.27, 130.55, 129.63, 128.71, 126.86, 126.54, 125.56, 124.13, 123.26, 121.85, 57.07, 16.59. ^{19}F NMR (376 MHz, $CDCl_3$, 298K), δ (ppm): -71.39, -73.23. HRMS (ESI-MS), calcd for ($C_{31}H_{25}N_4S^+$): m/z $[M]^+$: 485.1793; found, m/z 485.1795.

Supplementary results

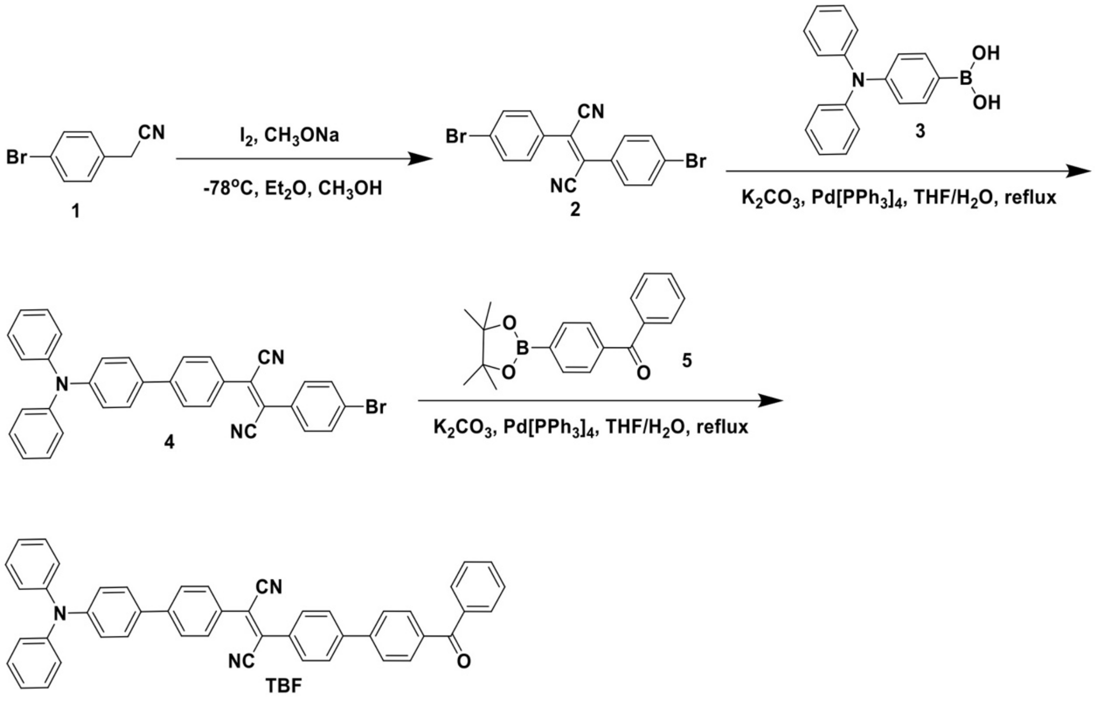

Fig. S1. The synthetic route to TBF.

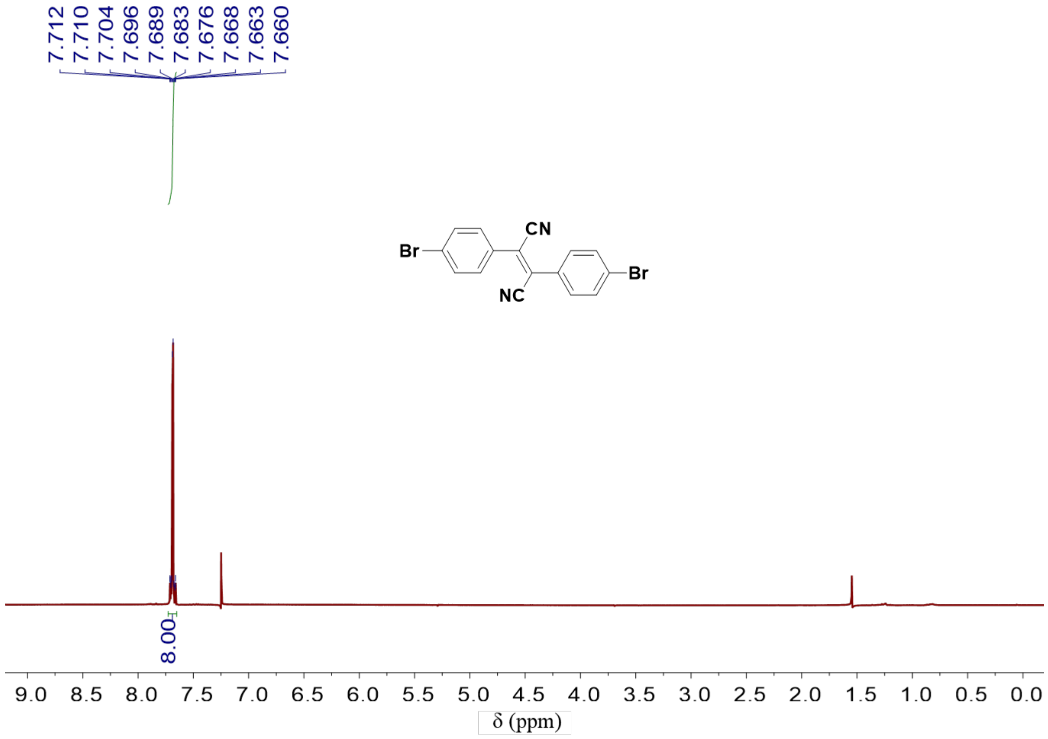

Fig. S2. ^{1}H NMR spectrum of compound 2 in chloroform-d.

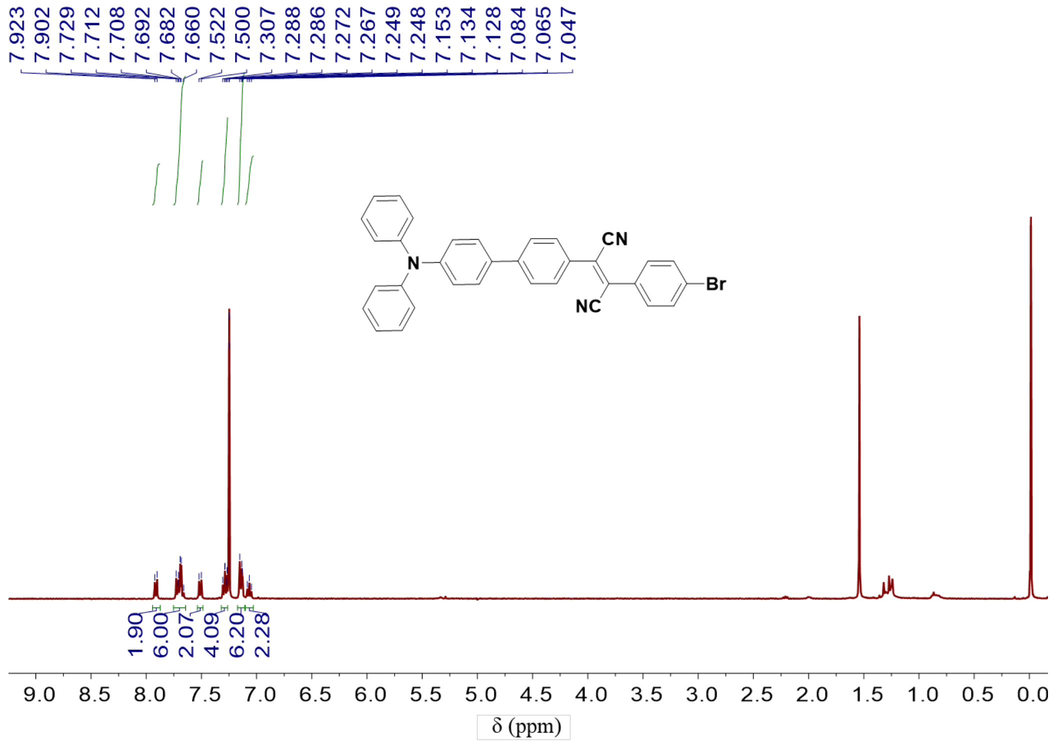

Figure S3. ^{1}H NMR spectrum of compound 4 in chloroform-d.

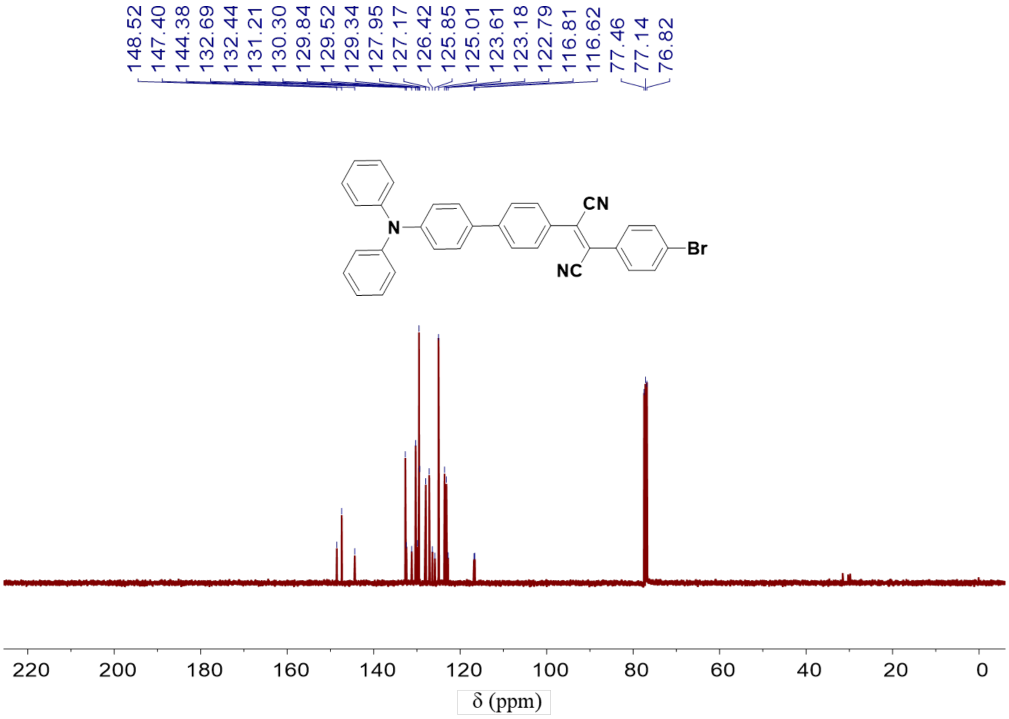

Figure S4. ^{13}C NMR spectrum of compound 4 in chloroform-d.

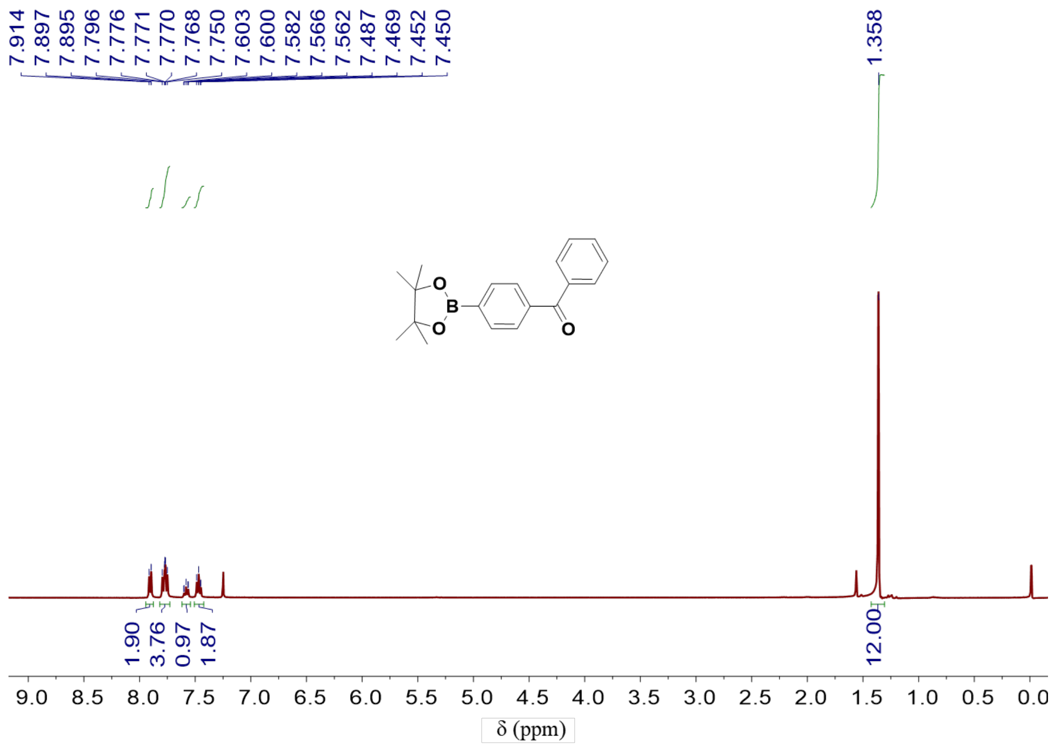

Figure S5. ^{1}H NMR spectrum of compound 5 in chloroform-d.

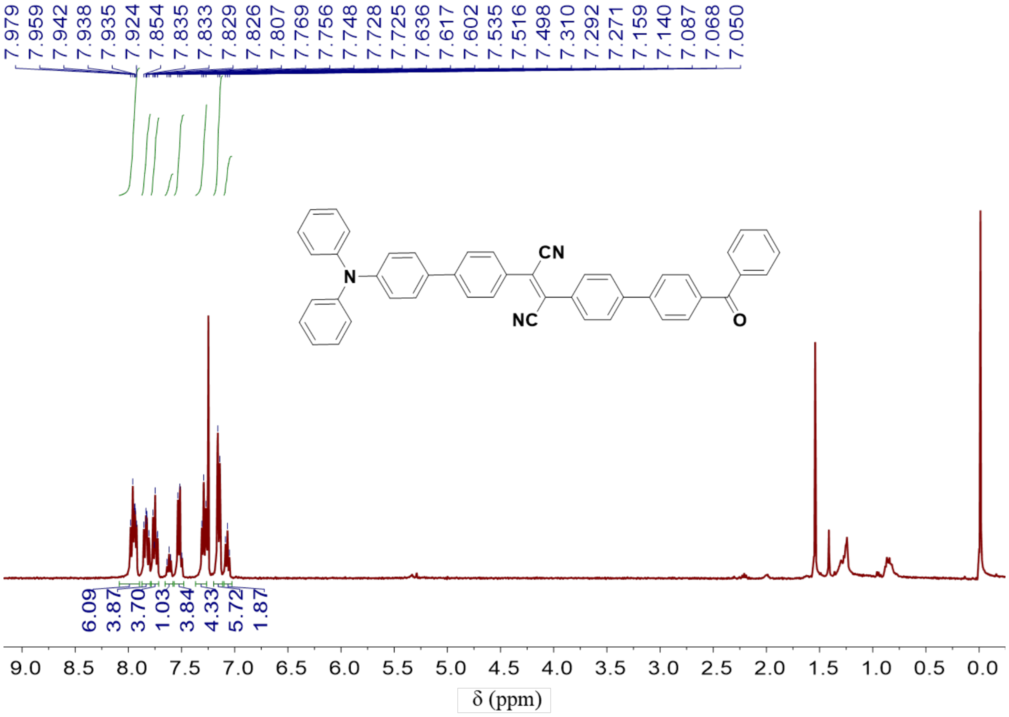

Figure S6. ^{1}H NMR spectrum of compound TBF in chloroform-d.

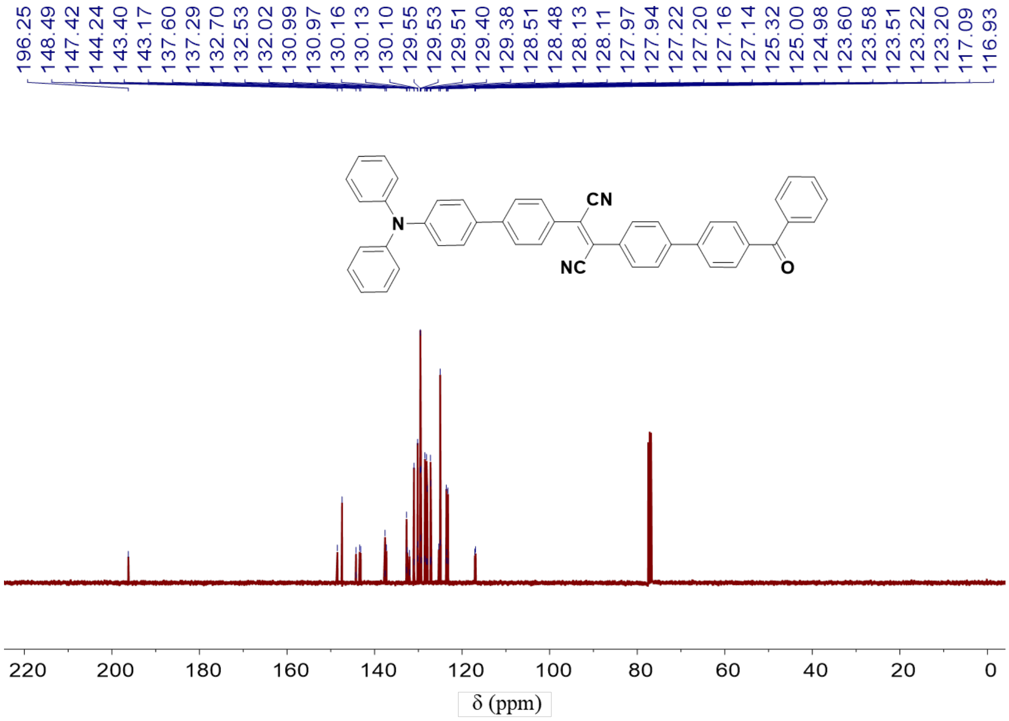

Figure S7. ^{13}C NMR spectrum of compound TBF in chloroform-d.

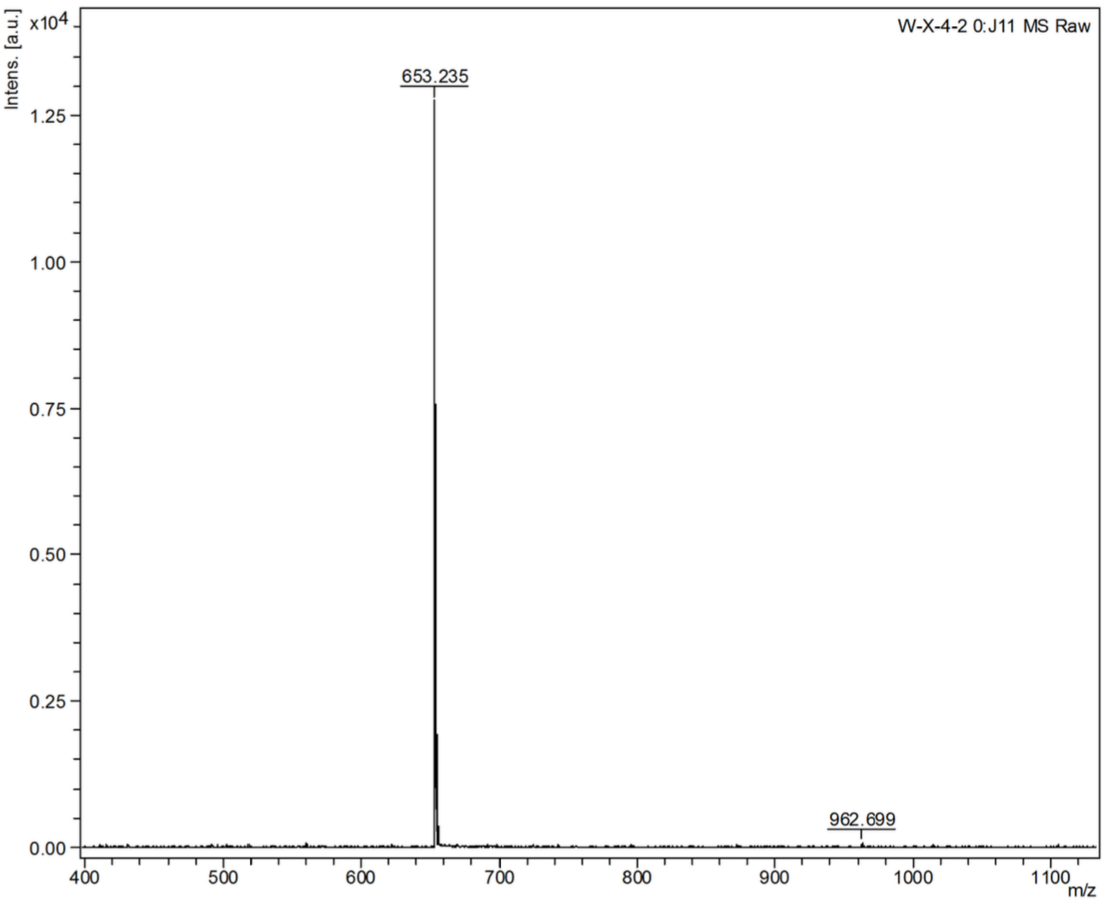

Figure S8. HRMS (MALDI-TOF) spectrum of compound TBF.

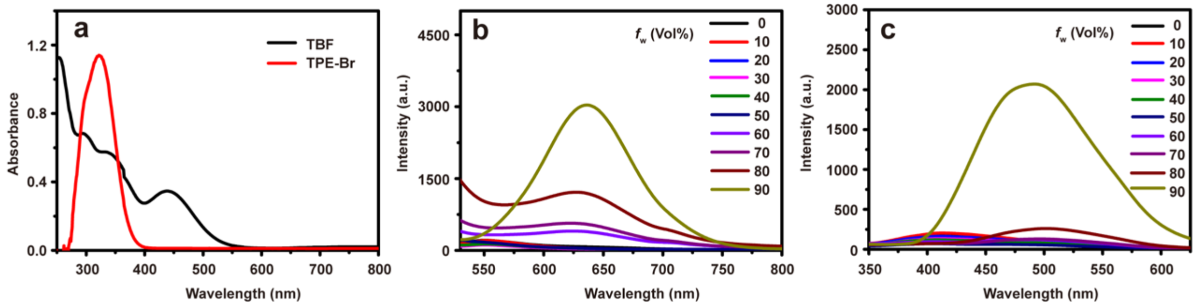

Figure S9. The spectra of TBF and TPE-Br. (a) The UV-vis absorbance spectra of TBF and TPE-Br in THF. Emission spectra of (b) TBF (Ex: 467 nm) and (c) TPE-Br (Ex: 320 nm) in THF/water mixture with different volume ratios. [TBF] = 10 μM, [TPE-Br] = 10 μM.

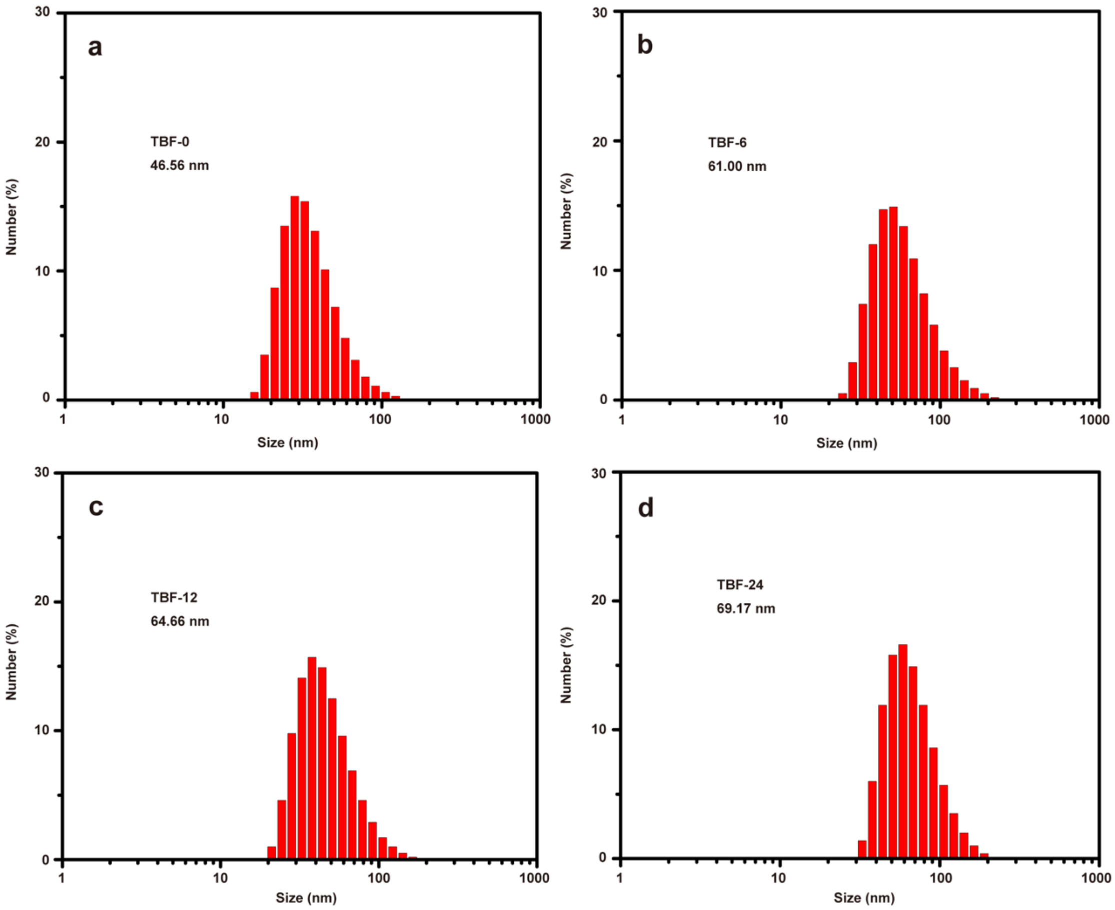

Figure S10. Hydrodynamic sizes. The DLS results of BNPs (TBF encapsulated with DSPE-mPEG) with different binary molecular ratios.

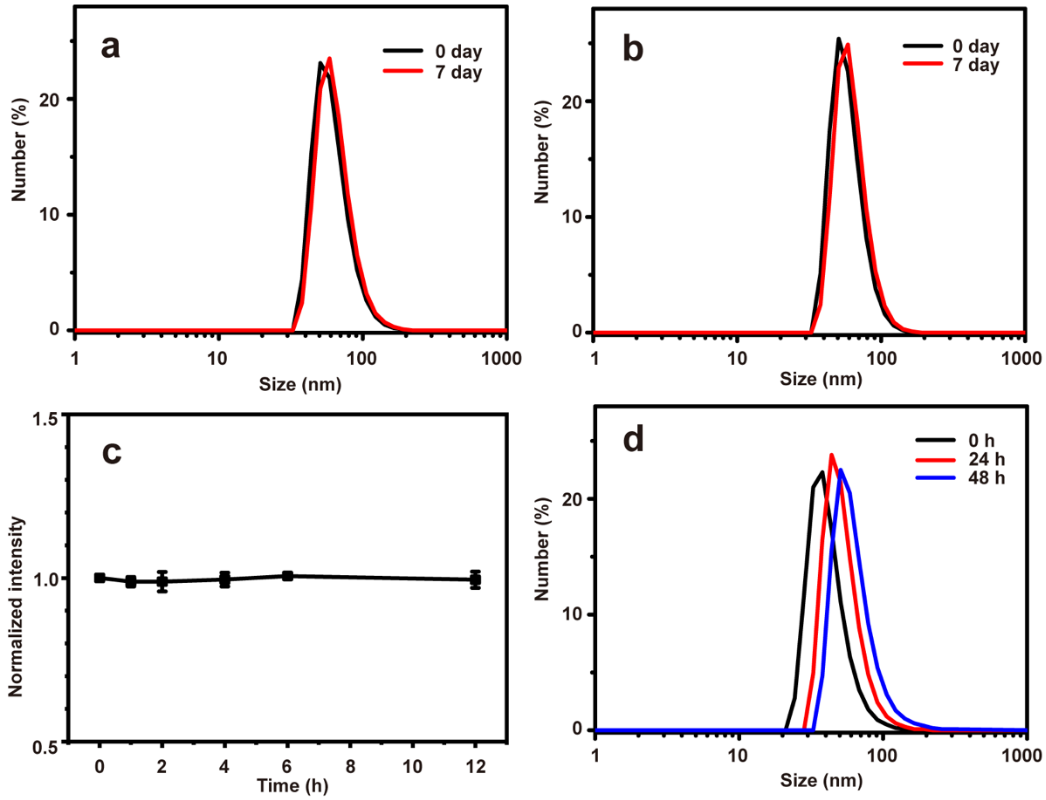

Figure S11. Long-term stability of BNPs. The stability of TBF-24 in (**a**) aqueous solution, (**b**) PBS, (**c**) DMEM medium with 10% FBS, and (**d**) TBF-36 in PBS.

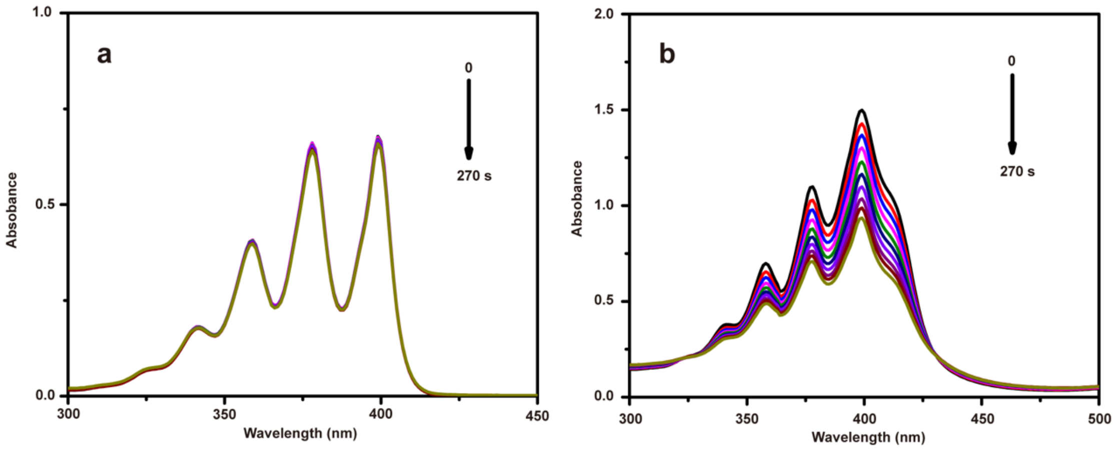

Figure S12. Absorption spectra. The spectra of (**a**) ABDA, and (**b**) ABDA with Ce6 (6.5 μM) mixture solution under light irradiation for different times.

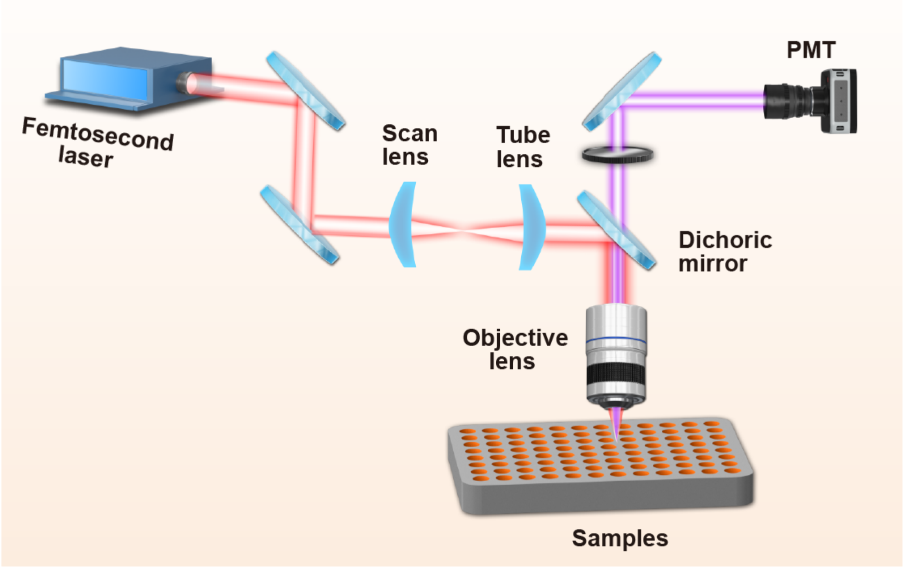

Figure S13. Experimental setups. Schematic of the home-built two-photon absorption and action cross-section measuring system.

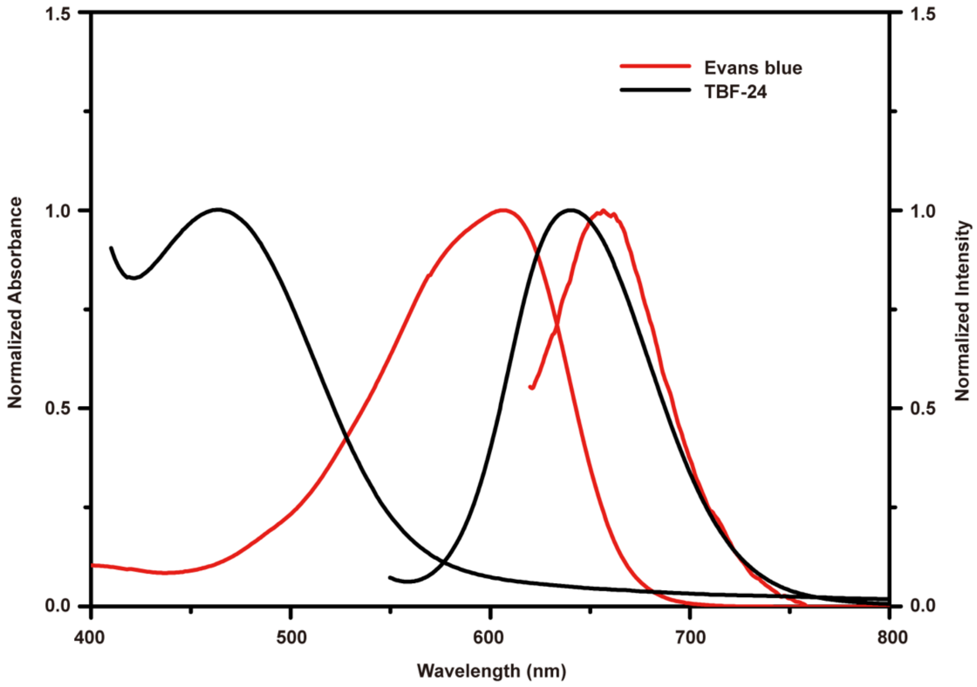

Figure S14. The spectra of TBF-24 and Evans blue. Normalized UV-vis absorption and emission spectra of TBF-24 and Evans blue in aqueous media.

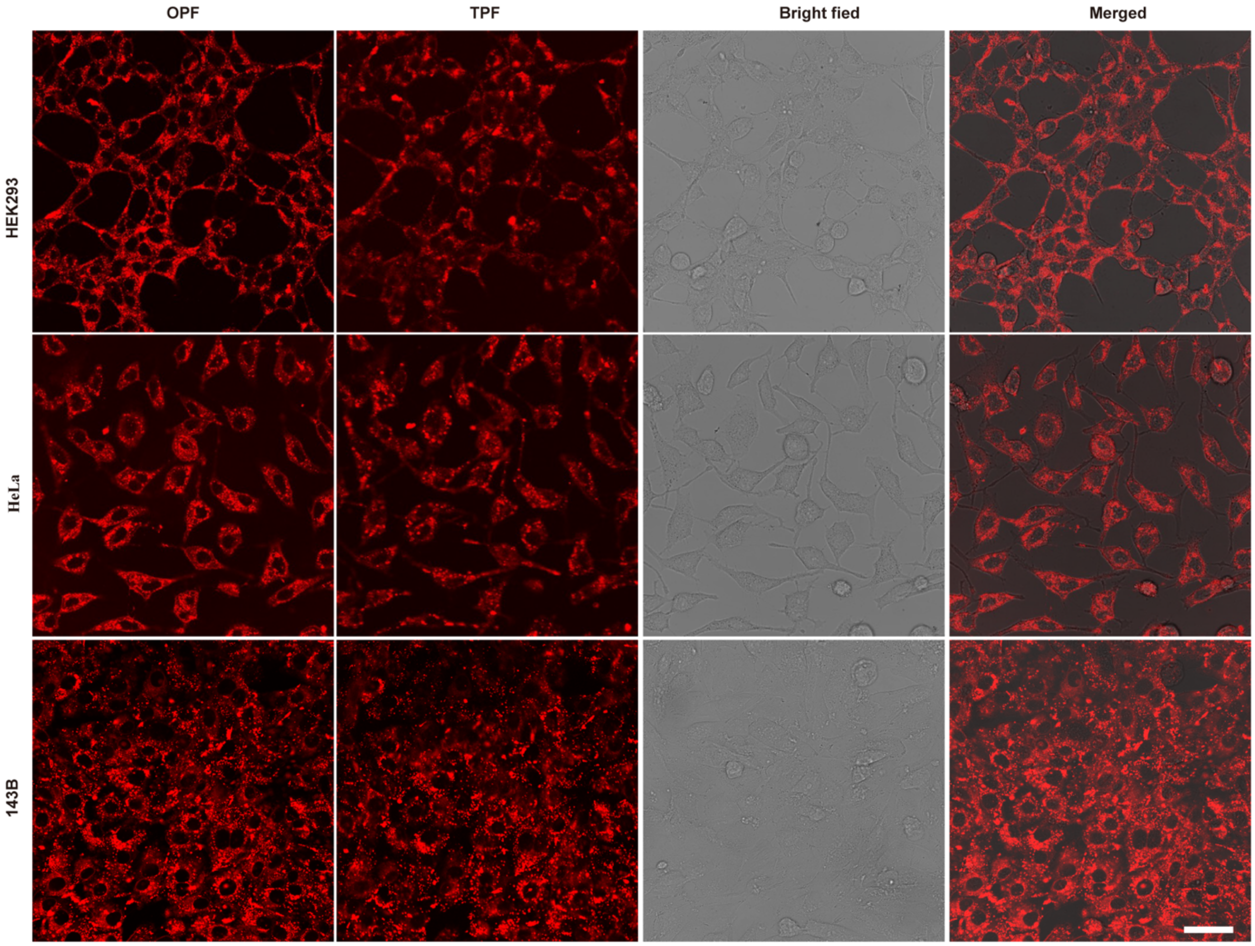

Figure S15. Cellular fluorescence imaging of TBF-24. One-photon fluorescence (OPF), two-photon fluorescence (TPF), bright field images, and the merged (OPF and bright field) of HEK293, HeLa, and 143B cells labeled with the TBF-24 (50 μM based on TBF). scale bar: 50 μm.

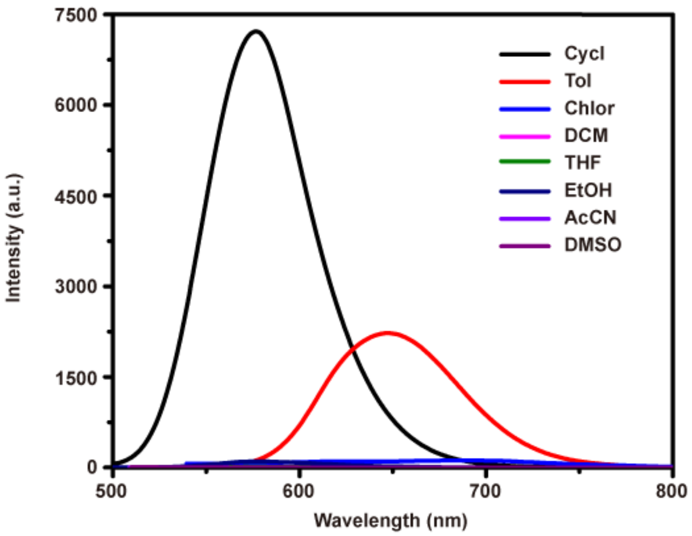

Figure S16. Fluorescence spectra of TBF molecular in different solvents. [TBF] = 5.0 μM, Ex: 467 nm. Cycl: cyclohexane; Tol: toluene; Chlor: chloroform, DCM: dichloromethane; THF: tetrahydrofuran; EtOH: ethanol; AcCN: acetonitrile; DMSO: dimethylsulfoxide.

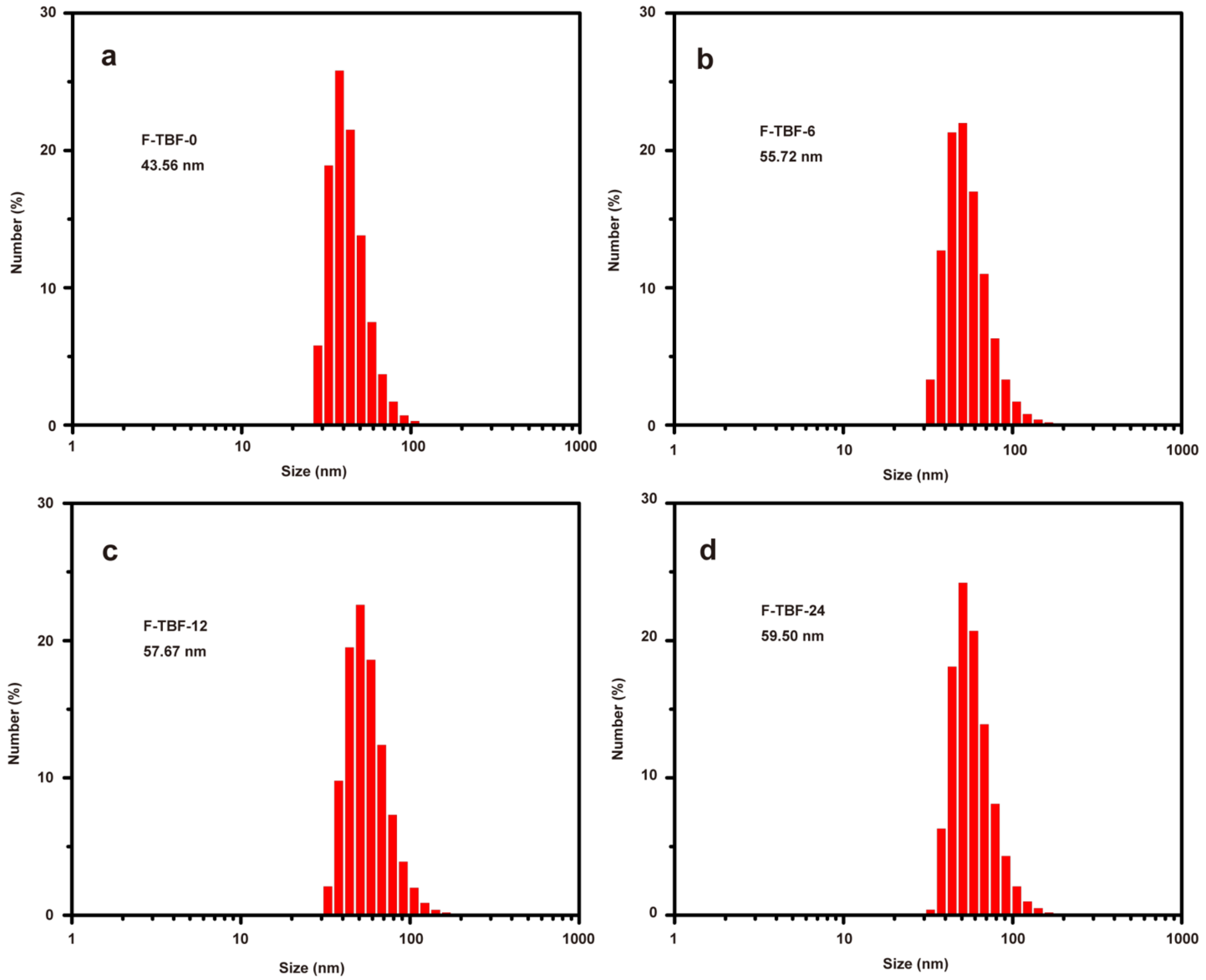

Figure S17. Hydrodynamic sizes of BNPs. The DLS results of F-TBF-0, F-TBF-6, F-TBF-12, and F-TBF-24 (TBF encapsulated with F127) with different binary molecular ratios.

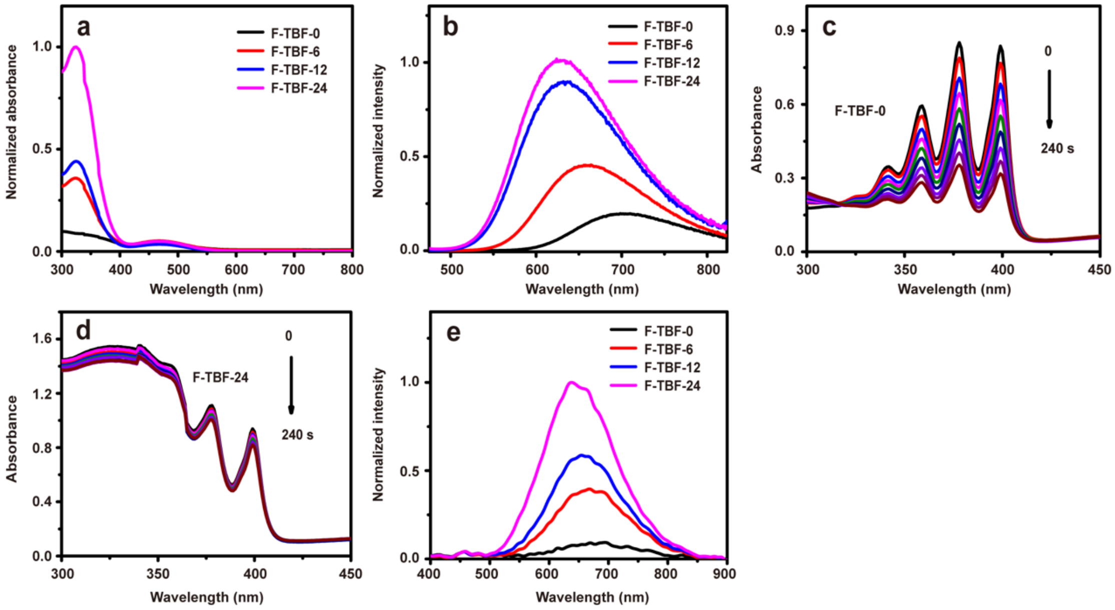

Figure S18. Photophysical properties of F-BNPs. (a) Normalized UV-vis absorption, (b) fluorescence spectra of F-TBF-0, F-TBF-6, F-TBF-12, and F-TBF-24 (TBF encapsulated with F127). Ex: 467 nm. The absorption spectra of the (c) ABDA and F-TBF-0, (d) ABDA and F-TBF-24 mixture solution under light irradiation (400-700 nm, 70 mW cm^{-2}) for different time. [F-TBF-0] = [F-BNPs] = 6.5 μM based on TBF, [ABDA] = 50 μM. (e) TPF spectra of F-TBF-0, F-TBF-6, F-TBF-12, F-TBF-24, Ex: 960 nm.

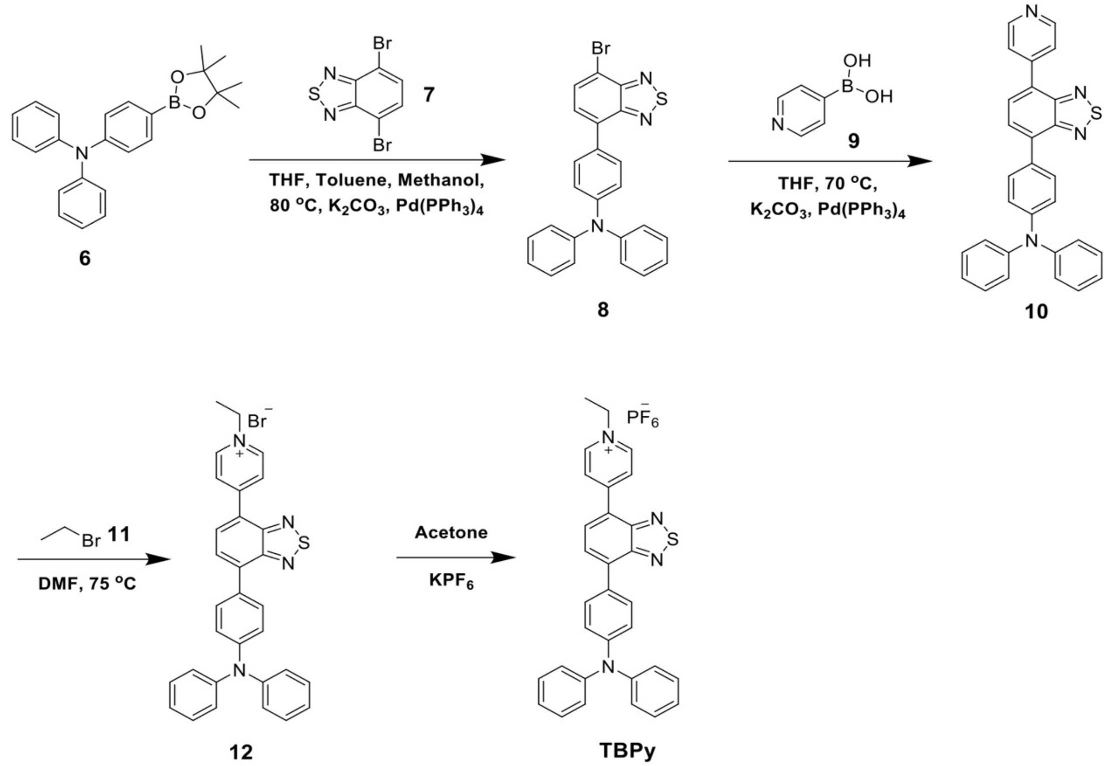

Fig. S19. The synthetic route to TBPy.

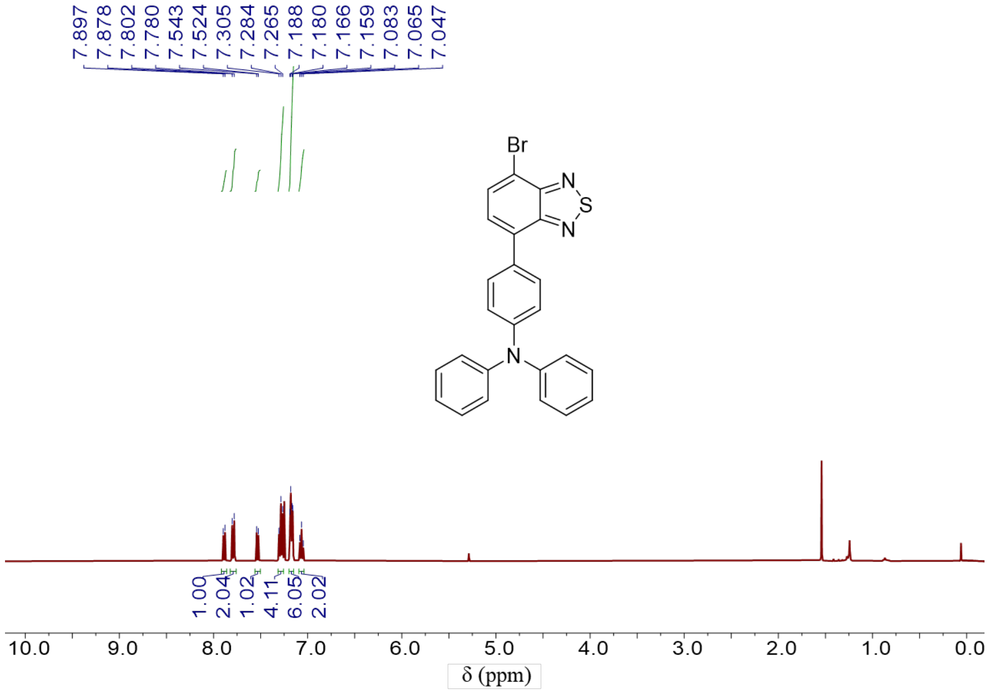

Figure S20. ^{1}H NMR spectrum of compound 8 in chloroform-d.

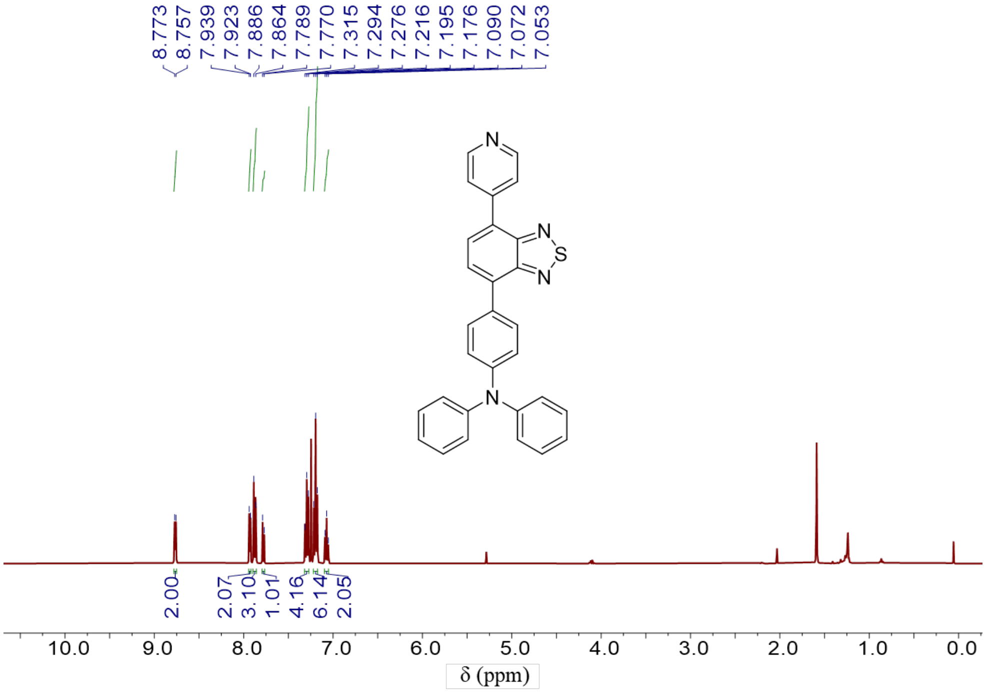

Figure S21. ^{1}H NMR spectrum of compound 10 in chloroform-d.

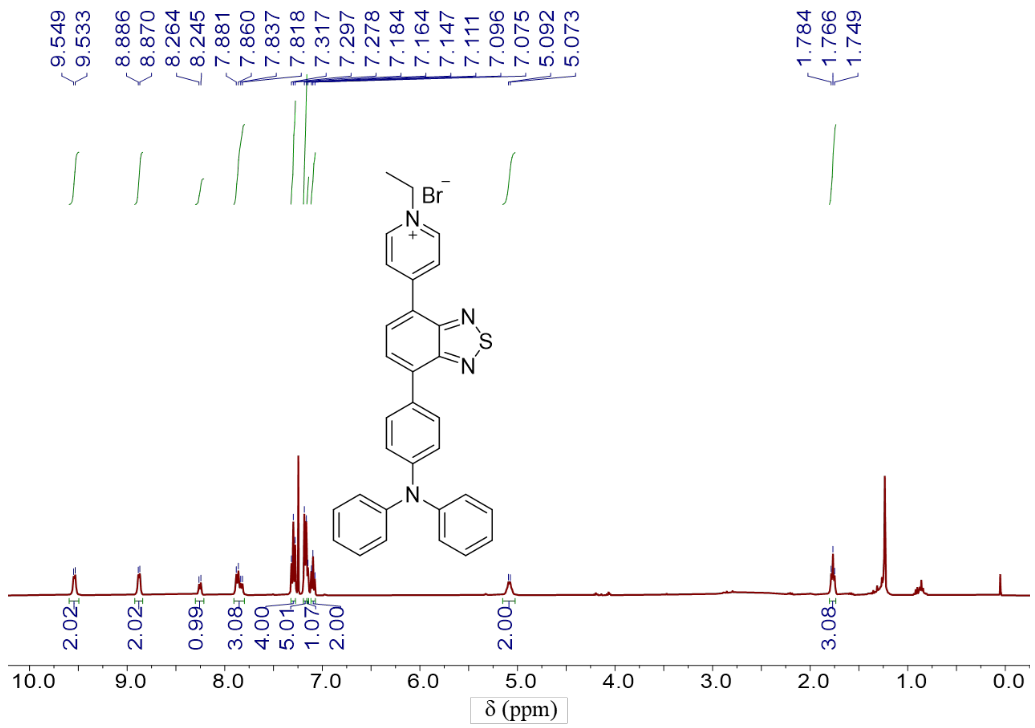

Figure S22. ^{1}H NMR spectrum of compound 12 in chloroform-d.

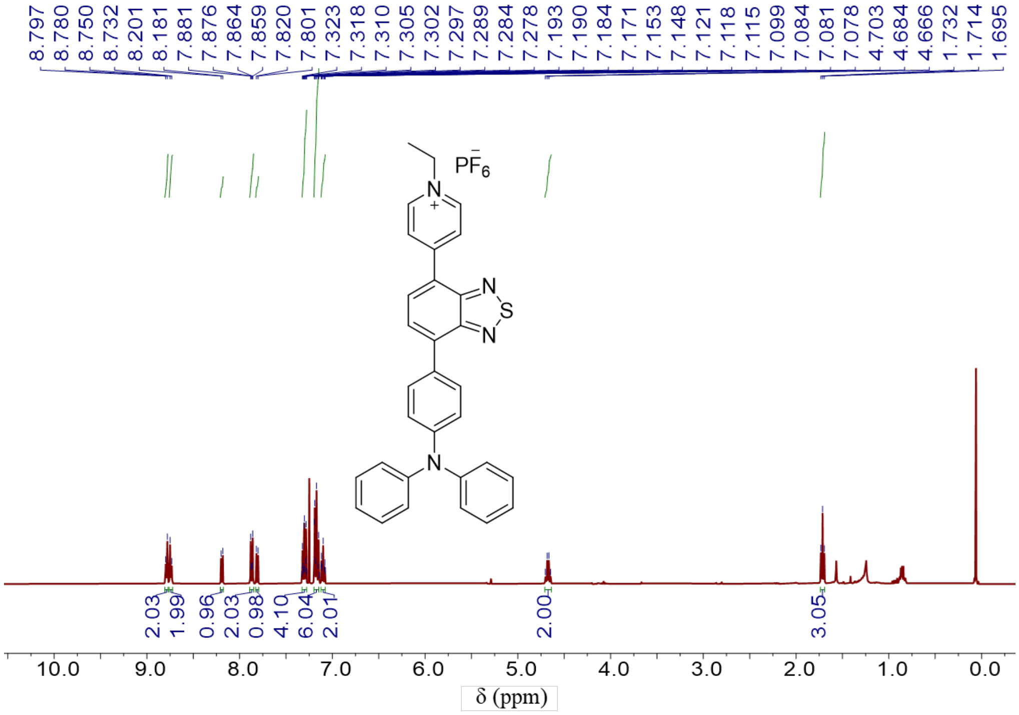

Figure S23. ^{1}H NMR spectrum of compound TBPy in chloroform-d.

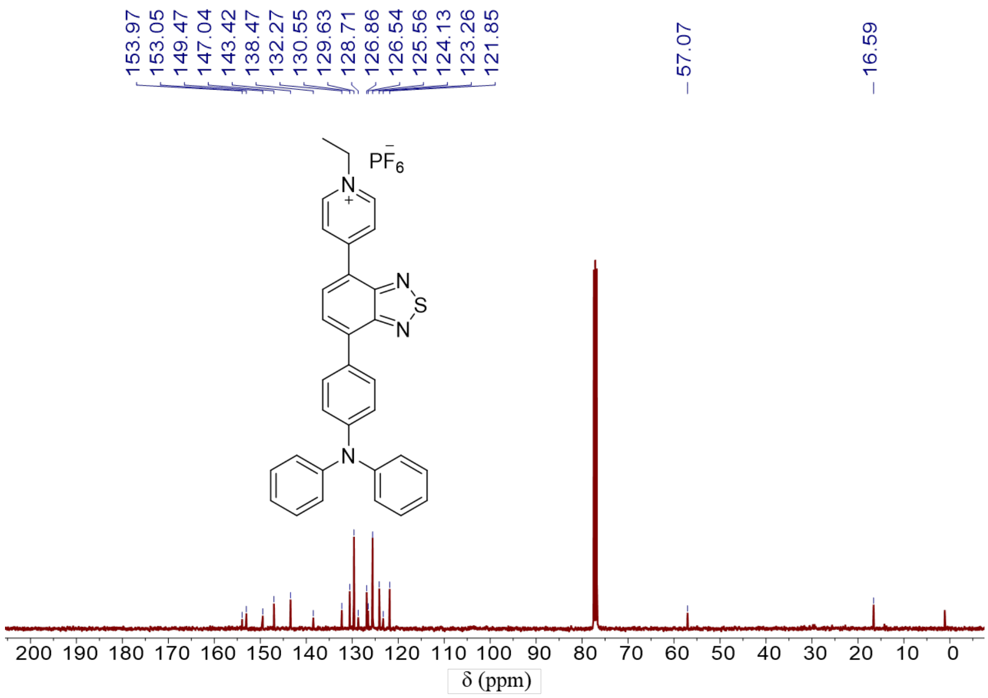

Figure S24. ^{13}C NMR spectrum of compound TBPy in chloroform-d.

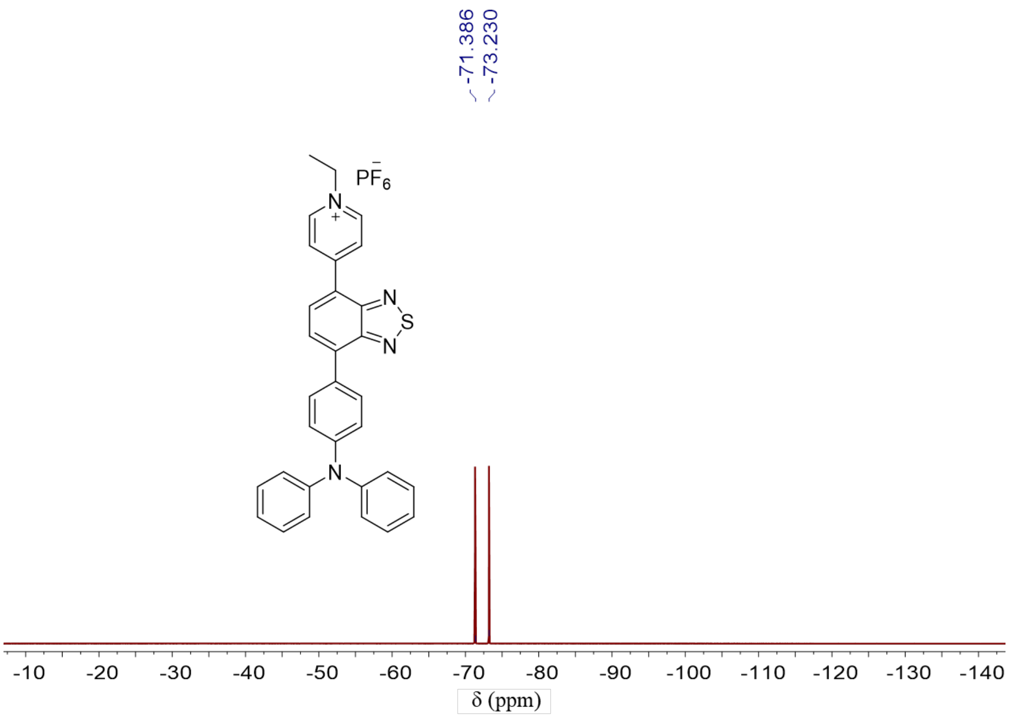

Figure S25. ^{19}F NMR spectrum of compound TBPy in chloroform-d.

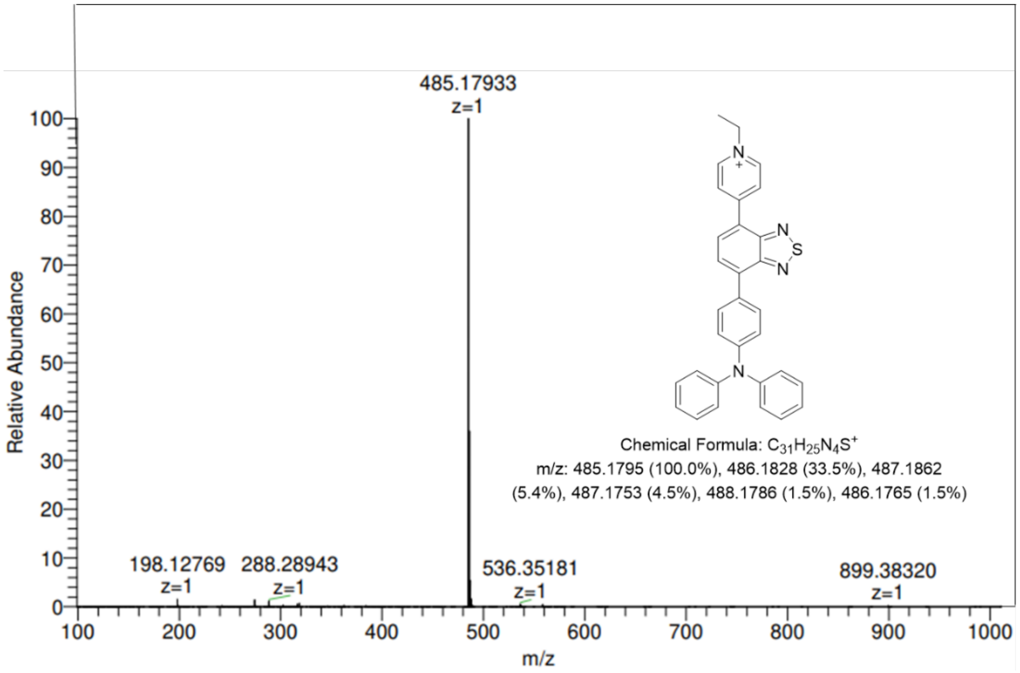

Figure S26. HRMS (ESI) spectrum of compound TBPy.

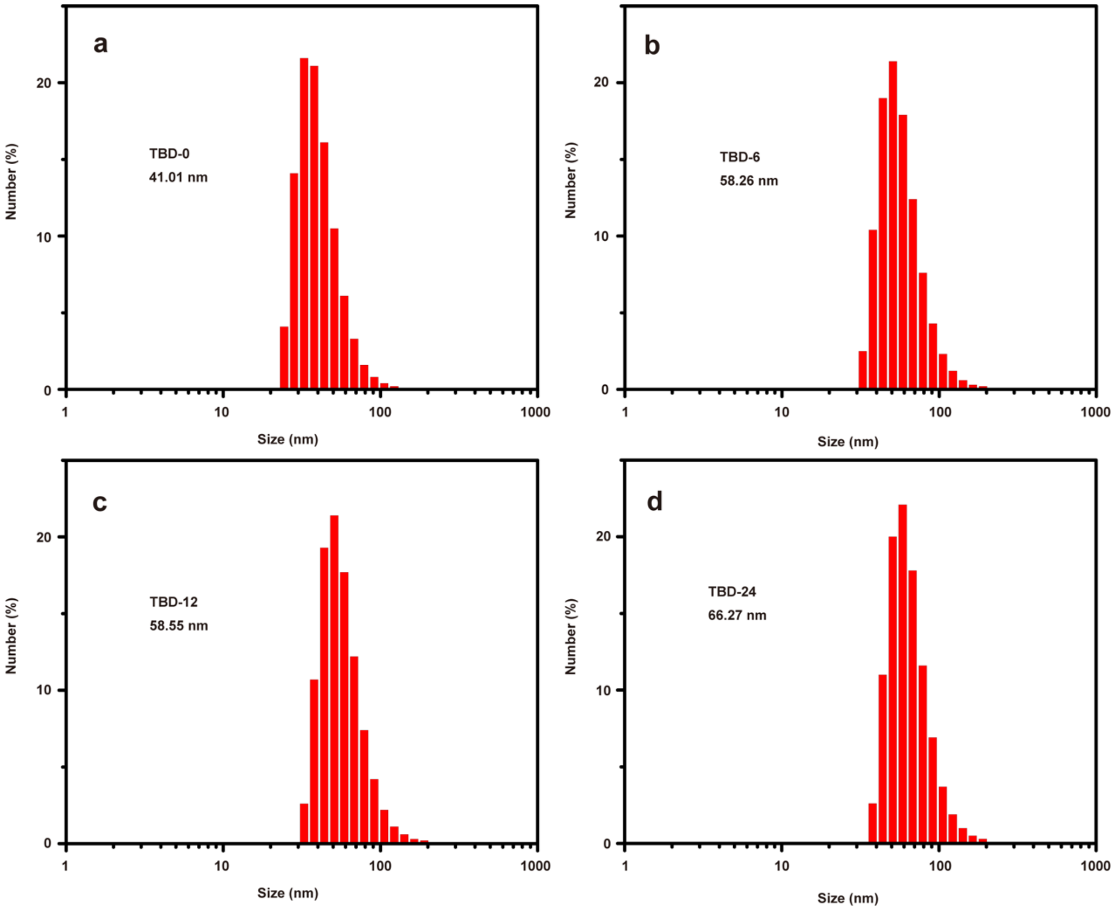

Figure S27. Hydrodynamic sizes of BNPs. The DLS results of TBD-0, TBD-6, TBD-12, and TBD-24 (TBD encapsulated with DSPE-mPEG) with different binary molecular ratios.

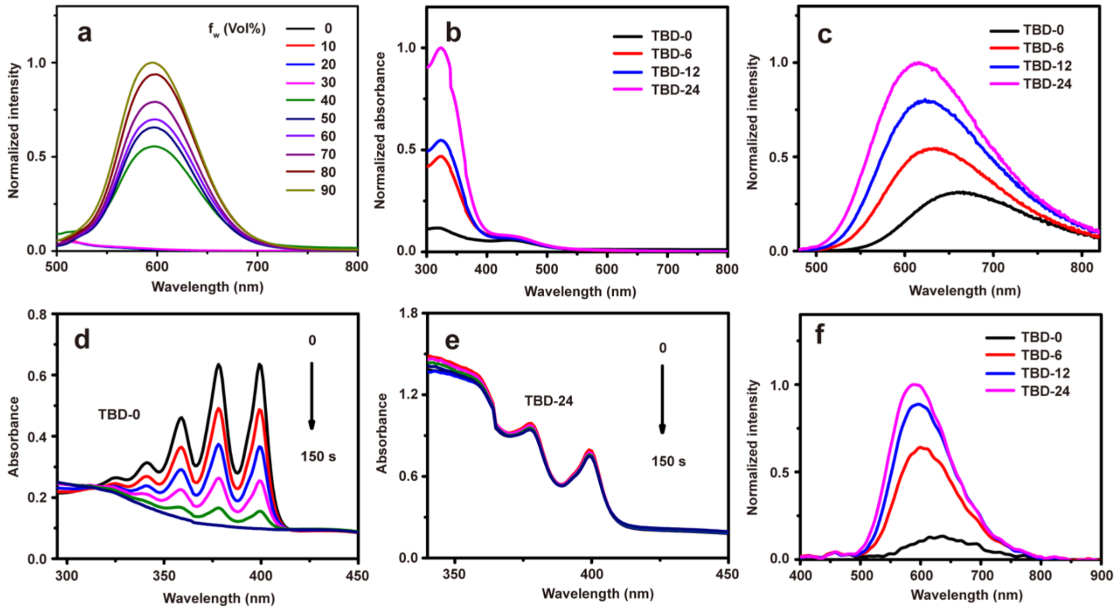

Figure S28. Photophysical properties of BNPs. (a) Emission spectra of TBD (Ex: 440 nm) in DMSO/water mixture solution with different volume ratios. [TBD] = 5 μM. (b) Normalized UV-vis absorption, (c) fluorescence emission spectra of TBD-0, TBD-6, TBD-12 and TBD-24. Ex: 440 nm. The absorption spectra of the (d) ABDA and TBD-0, (e) ABDA and TBD-24 mixture solution under light irradiation (400 - 700 nm, 70 mW cm^{-2}) for different time. [TBD-0] = [BNPs] = 6.5 μM based on TBD, [ABDA] = 50 μM. (f) Two-photon fluorescence spectra of TBD-0, TBD-6, TBD-12, and TBD-24 in aqueous solution. Ex: 960 nm.

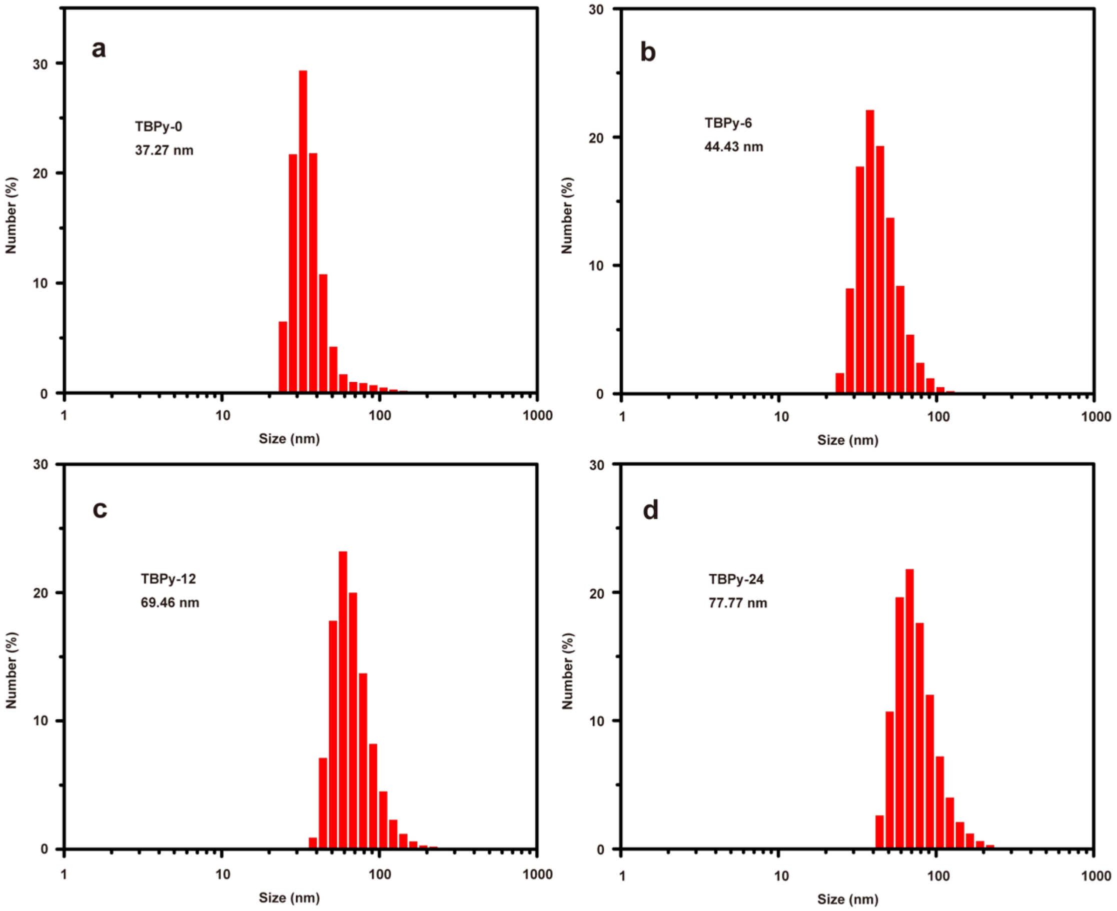

Figure S29. Hydrodynamic sizes of BNPs. The DLS results of TBPy-0, TBPy-6, TBPy-12, and TBPy-24 (TBPy encapsulated with DSPE-mPEG) with different binary molecular ratios.

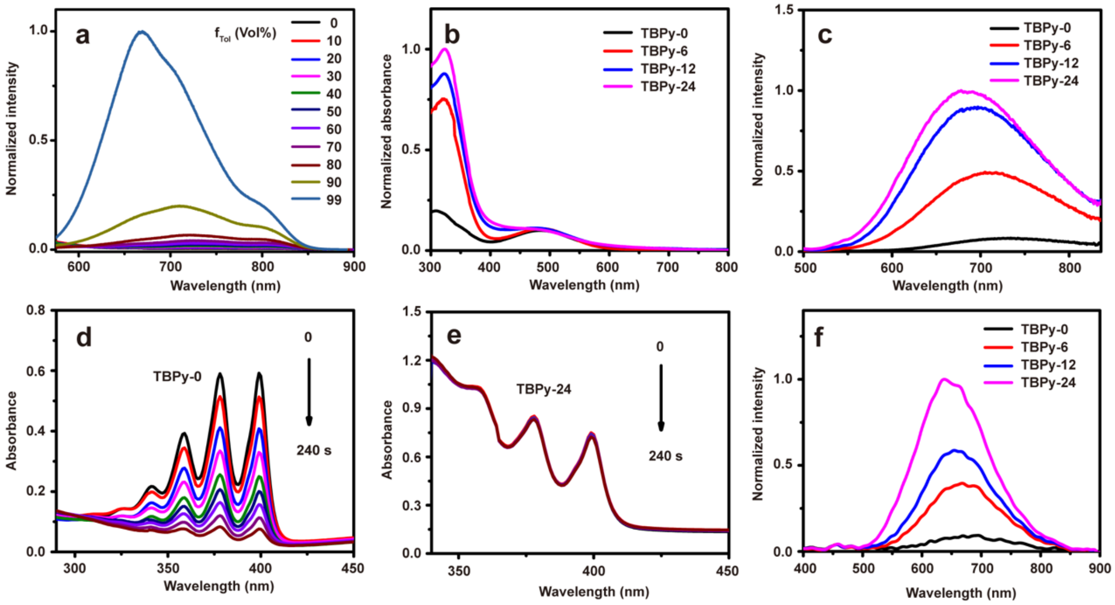

Figure S30. Photophysical properties. (a) Emission spectra of TBPy (Ex: 485 nm) in DMSO/Tol mixture solution with different volume ratios. [TBPy] = 10 μM. Tol: toluene. (b) Normalized UV-vis absorption, (c) fluorescence emission spectra of TBPy-0, TBPy-6, TBPy-12 and TBPy-24. Ex: 485 nm. The absorption spectra of the (d) ABDA and TBPy-0, (e) ABDA and TBPy-24 mixture solution under light irradiation (400 - 700 nm, 70 mW cm^{-2}) for different time. [TBPy-0] = [BNPs] = 6.5 μM based on TBPy, [ABDA] = 50 μM. (f) Two-photon fluorescence spectra of TBPy-0, TBPy-6, TBPy-12, and TBPy-24 in aqueous solution. Ex: 960 nm.

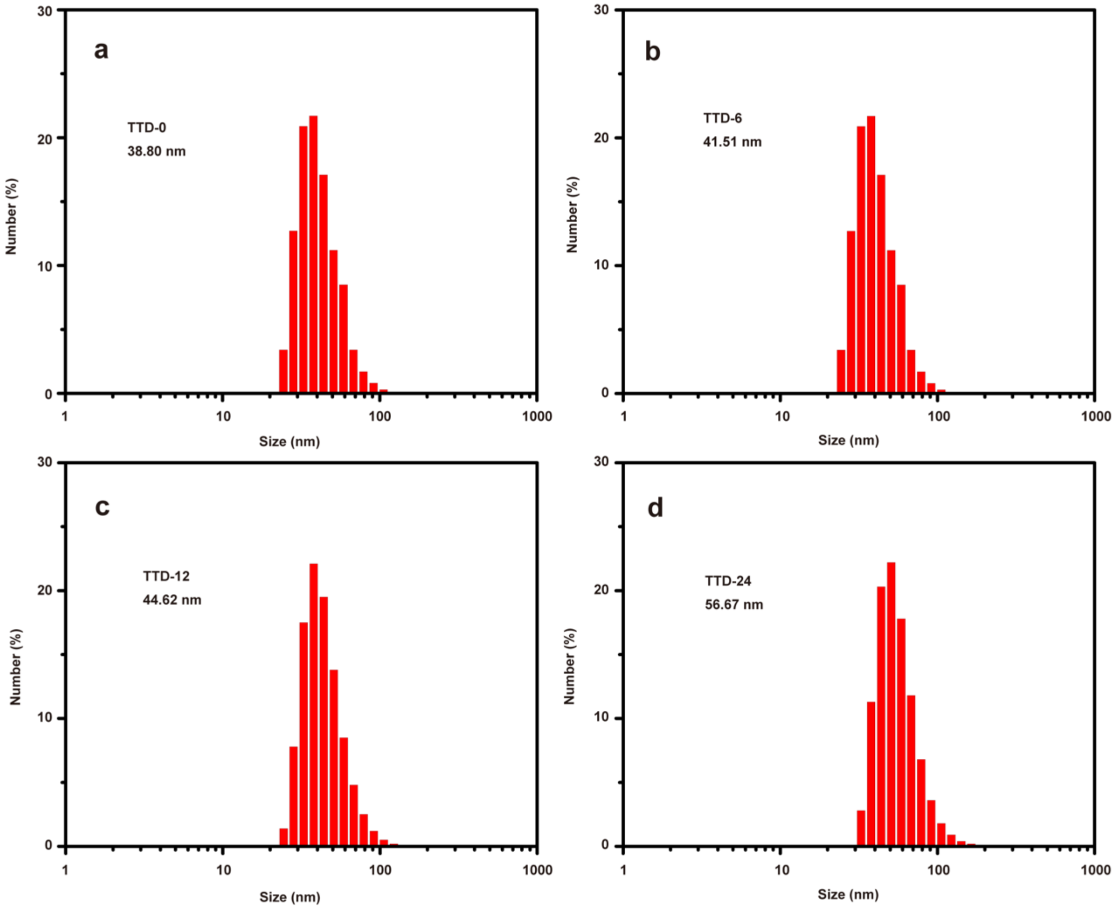

Figure S31. Hydrodynamic sizes of BNPs. The DLS results of TTD-0, TTD-6, TTD-12, and TTD-24 (TTD encapsulated with DSPE-mPEG) with different binary molecular ratios.

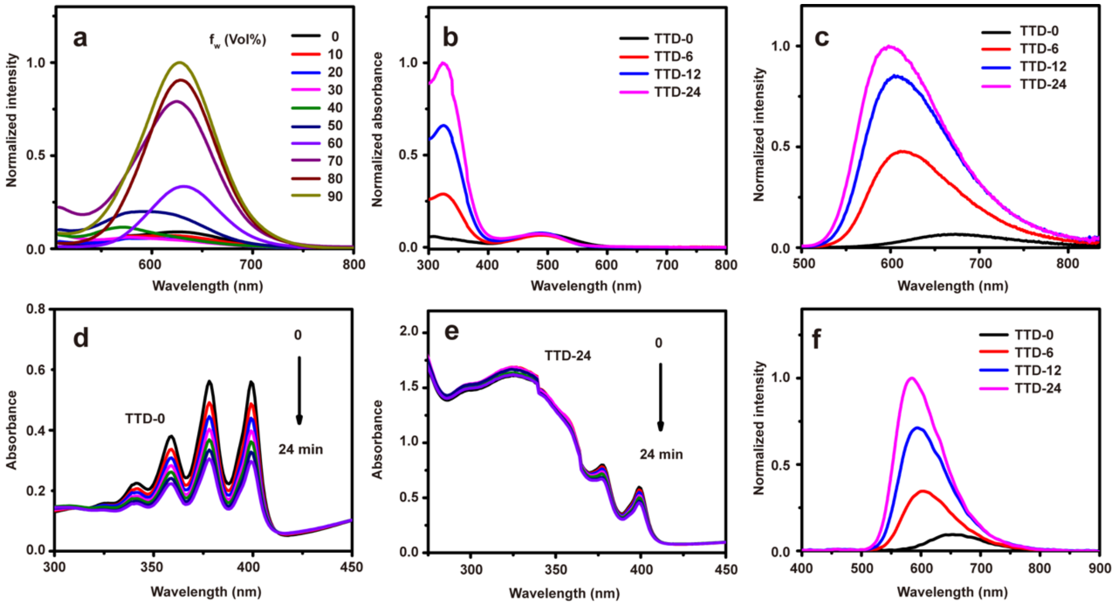

Figure S32. Photophysical properties. (a) Emission spectra of TTD (Ex: 488 nm) in DMSO/water mixture solution with different volume ratios. [TTD] = 5 μM. (b) Normalized UV-vis absorption, (c) fluorescence emission spectra of TTD-0, TTD-6, TTD-12 and TTD-24. Ex: 488 nm. The absorption spectra of the (d) ABDA and TTD-0, (e) ABDA and TTD-24 mixture solution under light irradiation (400 - 700 nm, 70 mW cm^{-2}) for different time. [TTD-0] = [BNPs] = 6.5 μM based on TTD, [ABDA] = 50 μM. (f) Two-photon fluorescence spectra of TTD-0, TTD-6, TTD-12, and TTD-24 in aqueous solution. Ex: 960 nm.

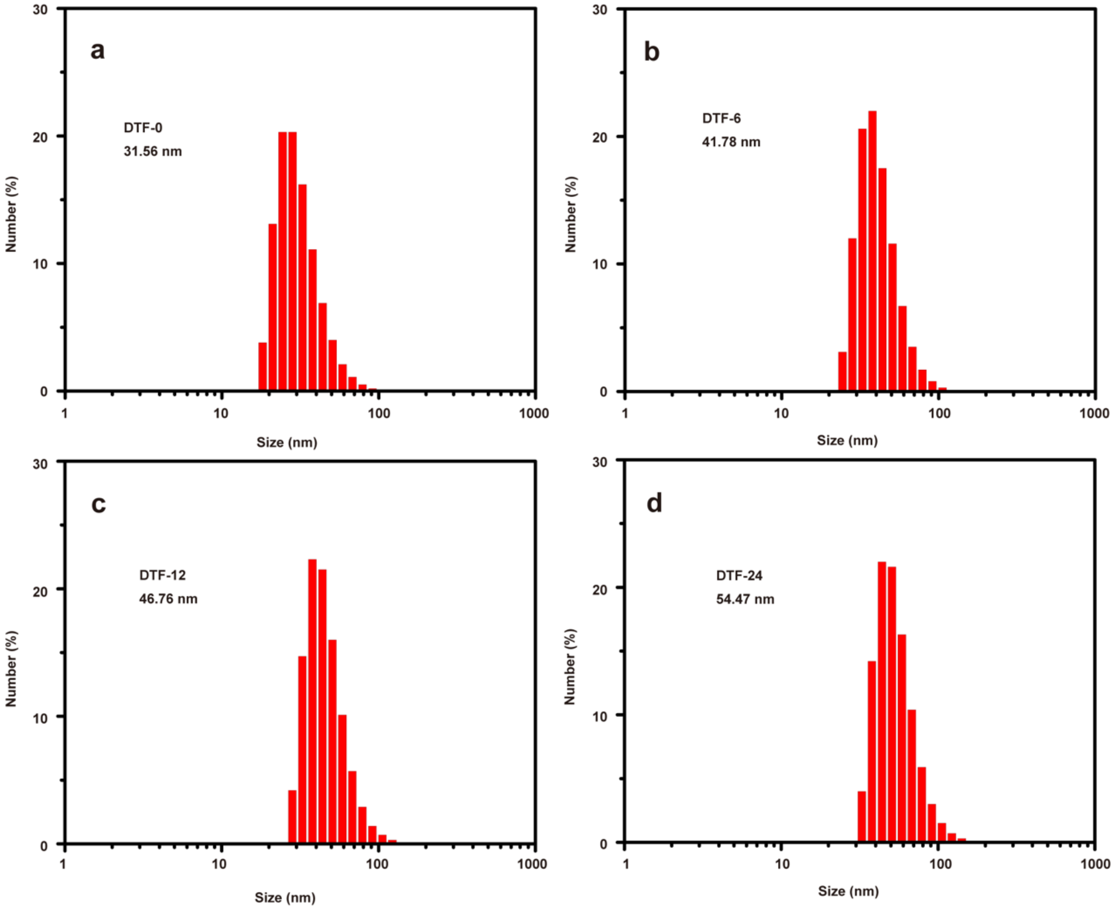

Figure S33. Hydrodynamic sizes of BNPs. The DLS results of DTF-0, DTF-6, DTF-12, and DTF-24 (DTF encapsulated with DSPE-mPEG) with different binary molecular ratios.

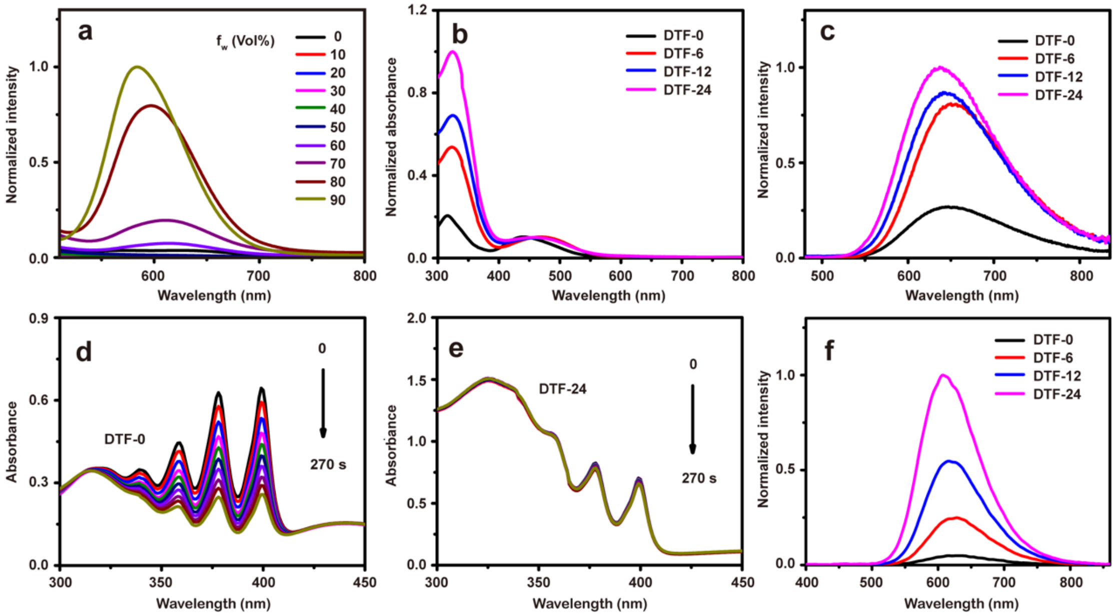

Figure S34. Photophysical properties. (a) Emission spectra of DTF (Ex: 441 nm) in THF/water mixture solution with different volume ratios. [DTF] = 5 μM. (b) Normalized UV-vis absorption, (c) fluorescence emission spectra of DTF-0, DTF-6, DTF-12 and DTF-24. Ex: 441 nm. The absorption spectra of the (d) ABDA and DTF-0, (e) ABDA and DTF-24 mixture solution under light irradiation (400 - 700 nm, 70 mW cm^{-2}) for different time. [DTF-0] = [BNPs] = 6.5 μM based on DTF, [ABDA] = 50 μM. (f) Two-photon fluorescence spectra of DTF-0, DTF-6, DTF-12, and DTF-24 in aqueous solution. Ex: 900 nm.

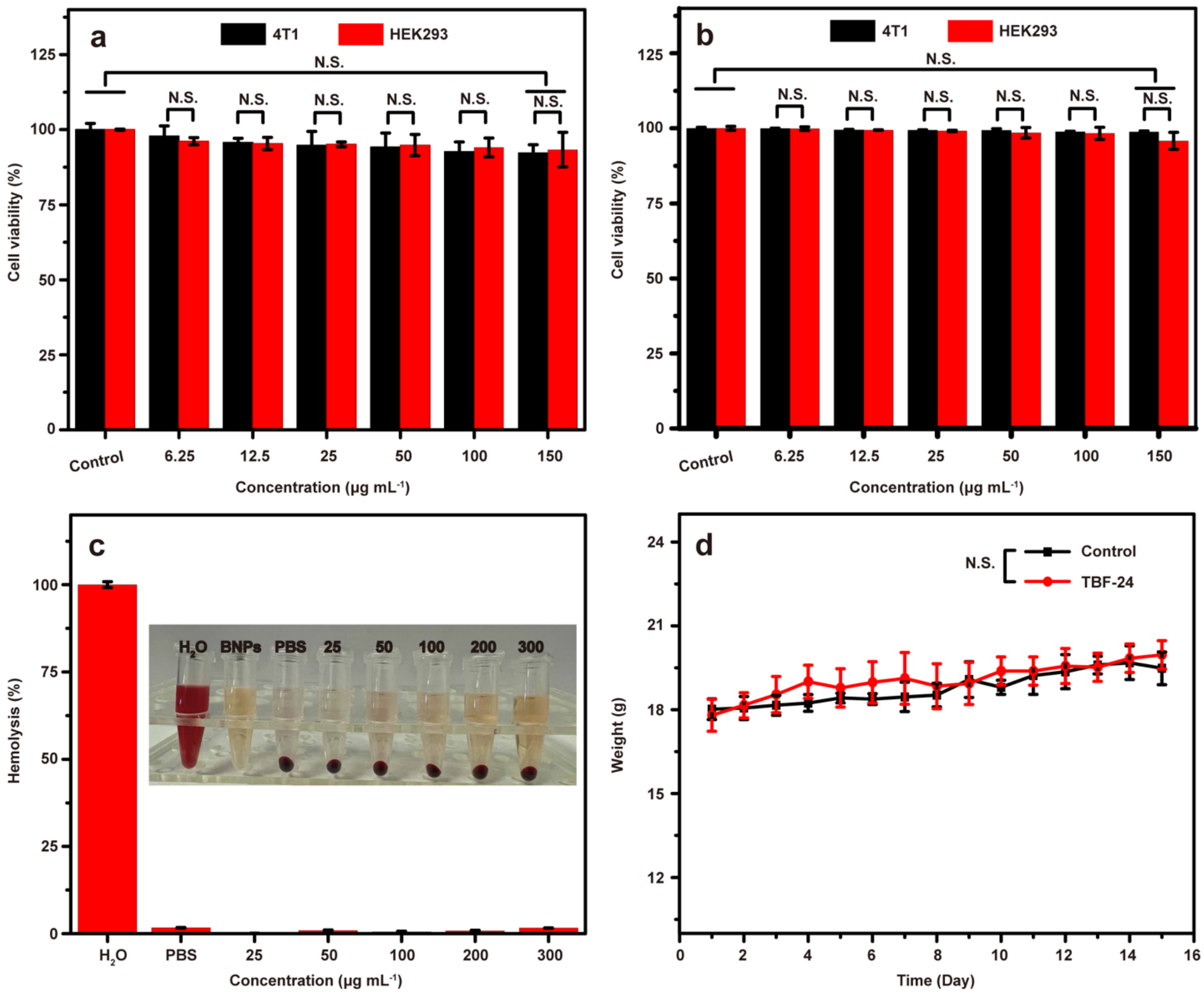

Figure S35. Biocompatibility evaluation. Cell viability of 4T1 cancer cells and HEK293 normal cells treated with various concentrations of TBF-24 for (a) 24 and (b) 48 h. Data were expressed as means ± s.d. (n = 3). (c) Hemolysis percentage of RBCs (2%, v/v) at various concentrations of TBF-24, inset photos: RBCs cultured with H_2O, TBF-24 (300 μg mL^{-1}, without RBCs), PBS, various concentrations of TBF-24 for 12 h. (d) Body weight variations of mice treated with TBF-24 or PBS for different time. N.S.: no significant difference.

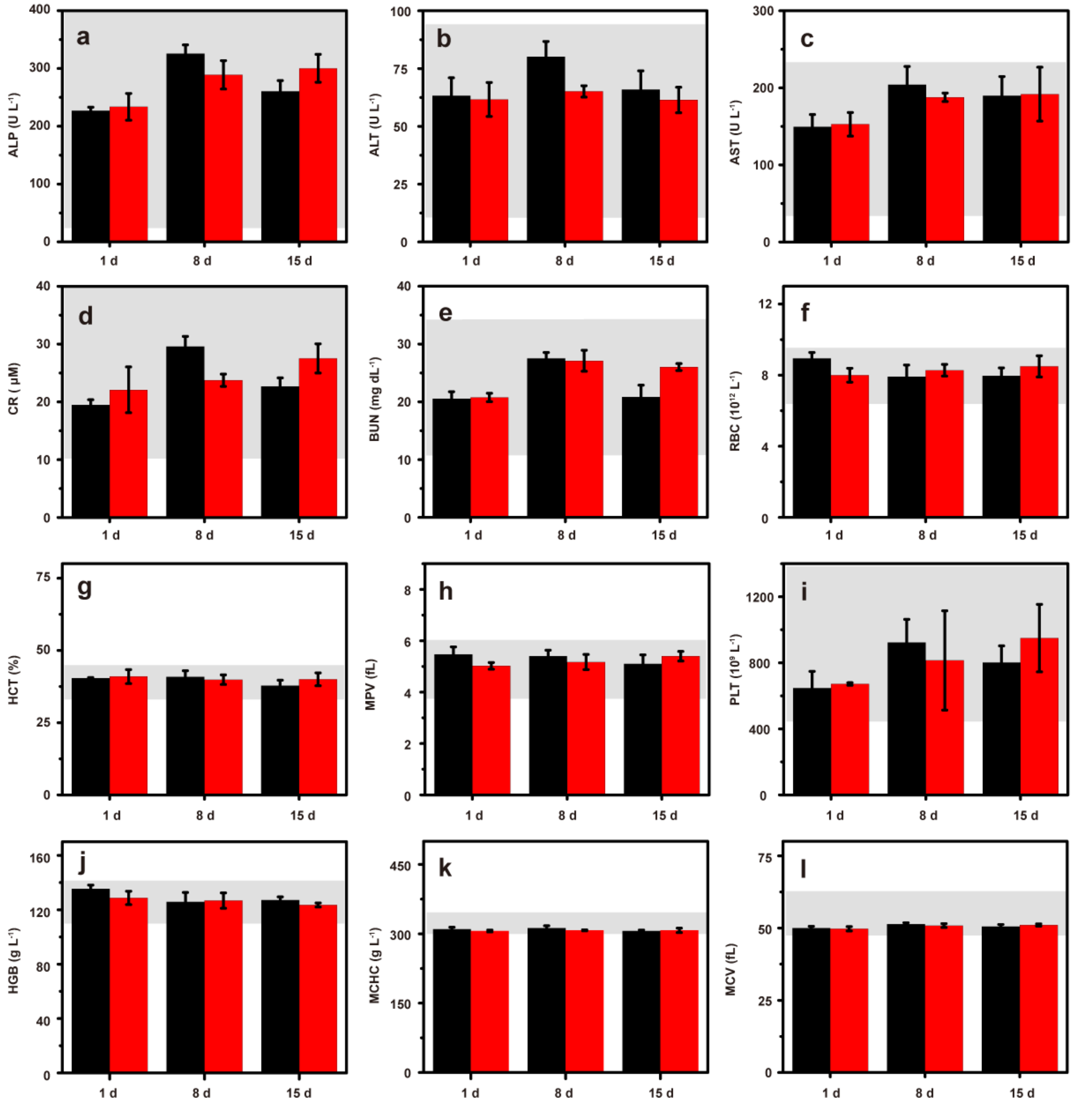

Fig. S36. Evaluation of blood indicators. (**a-e**) Blood biochemistry tests and (**f-l**) hematology parameters of untreated mice (black), and mice treated with TBF-24 (red) at day 1, 8, 15. [TBF-24] = 5 mg kg^{-1}. The reference range was marked in grey. Data were expressed as means ± s.d. (n = 3).

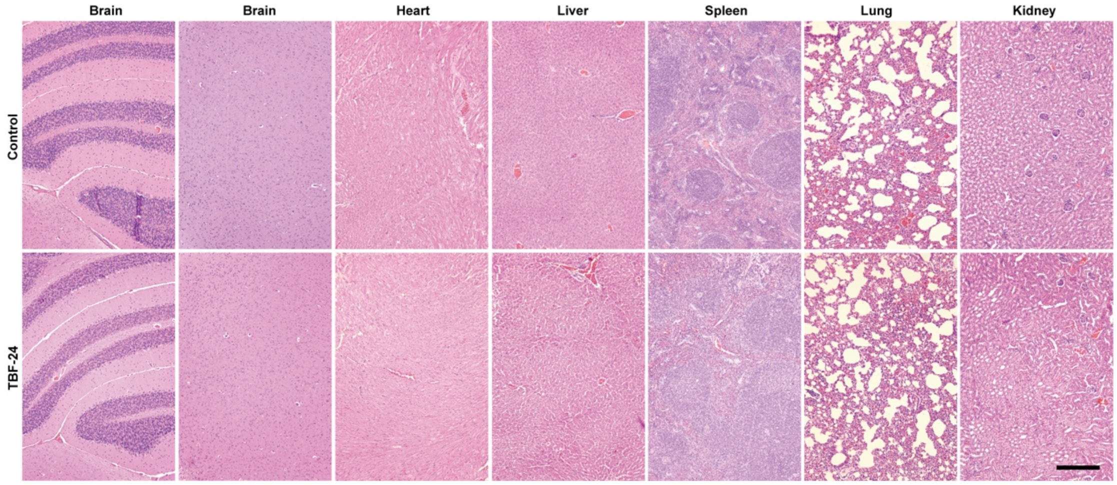

Figure S37. Histological examination. Mice's major organs (brain, heart, liver, spleen, lung, and kidney) are stained with hematoxylin and eosin (H&E). Tissues were collected from mice on day 15th after the administration of TBF-24 (5 mg kg^{-1}). The control mice were treated with PBS. Scale bar: 150 μm.

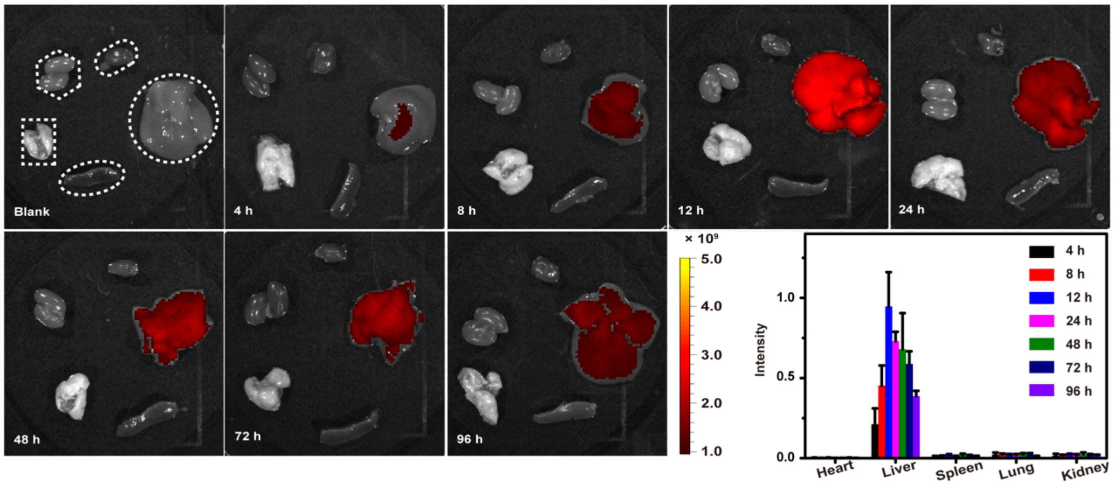

Figure S38. Biodistribution and metabolism of administrated TBF-24. Time-sequenced ex vivo fluorescence images and normalized fluorescence intensity of major organs excised from TBF-24 treated mice (5 mg kg^{-1}) and blank one treated with PBS. Data were expressed as means ± s.d. (n = 4). The oval, circle, olive shape, rectangle, and pentagon indicated the heart, liver, spleen, lung, and kidney, respectively.

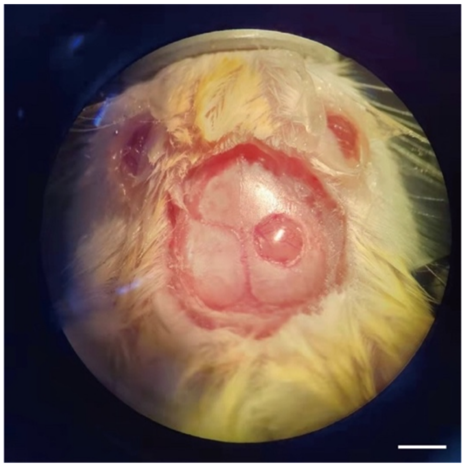

Figure S39. The photograph of the mice's cranial window. The cranial window (diameter, 5 mm) opened up through microsurgery for cerebral vascular imaging. Scale bar: 5 mm.

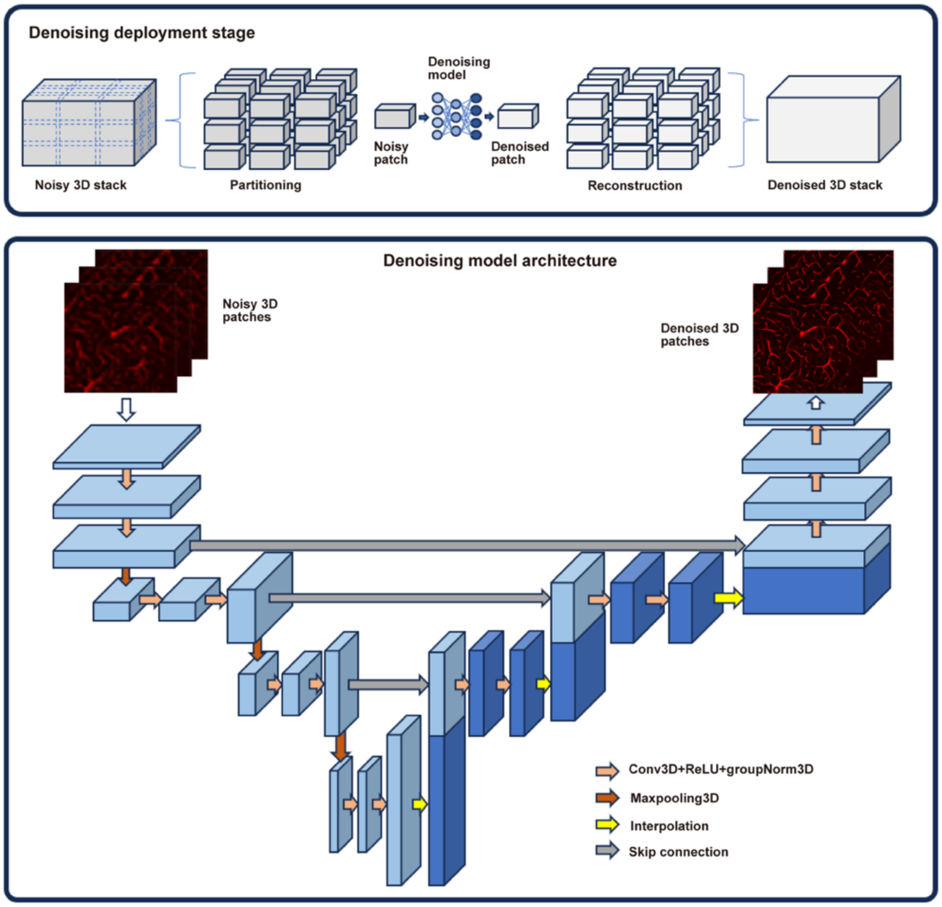

Figure S40. The process of deep learning algorithm denoising. Denoising deployment stage pipeline and self-supervising denoising model architecture.

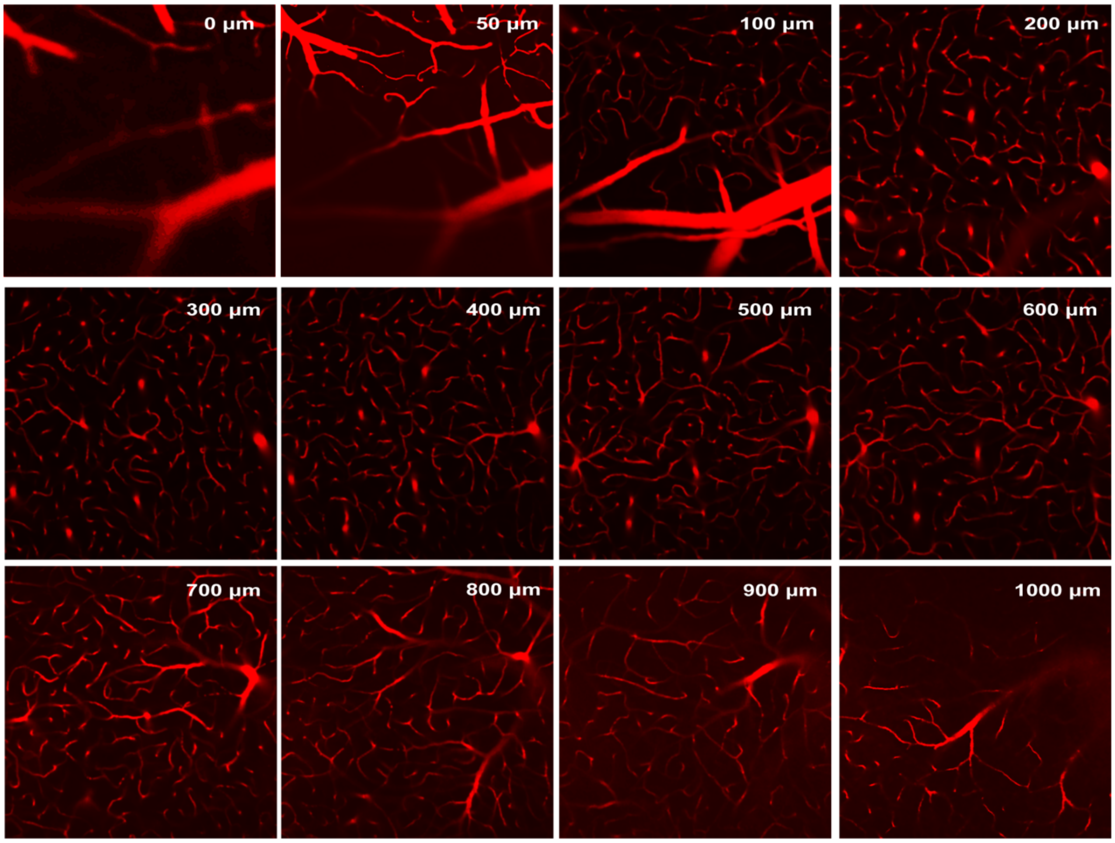

Figure S41. Denoising TPF images. The denoising TPF images of mouse cerebrovascular at different vertical depths (0-1.0 mm).

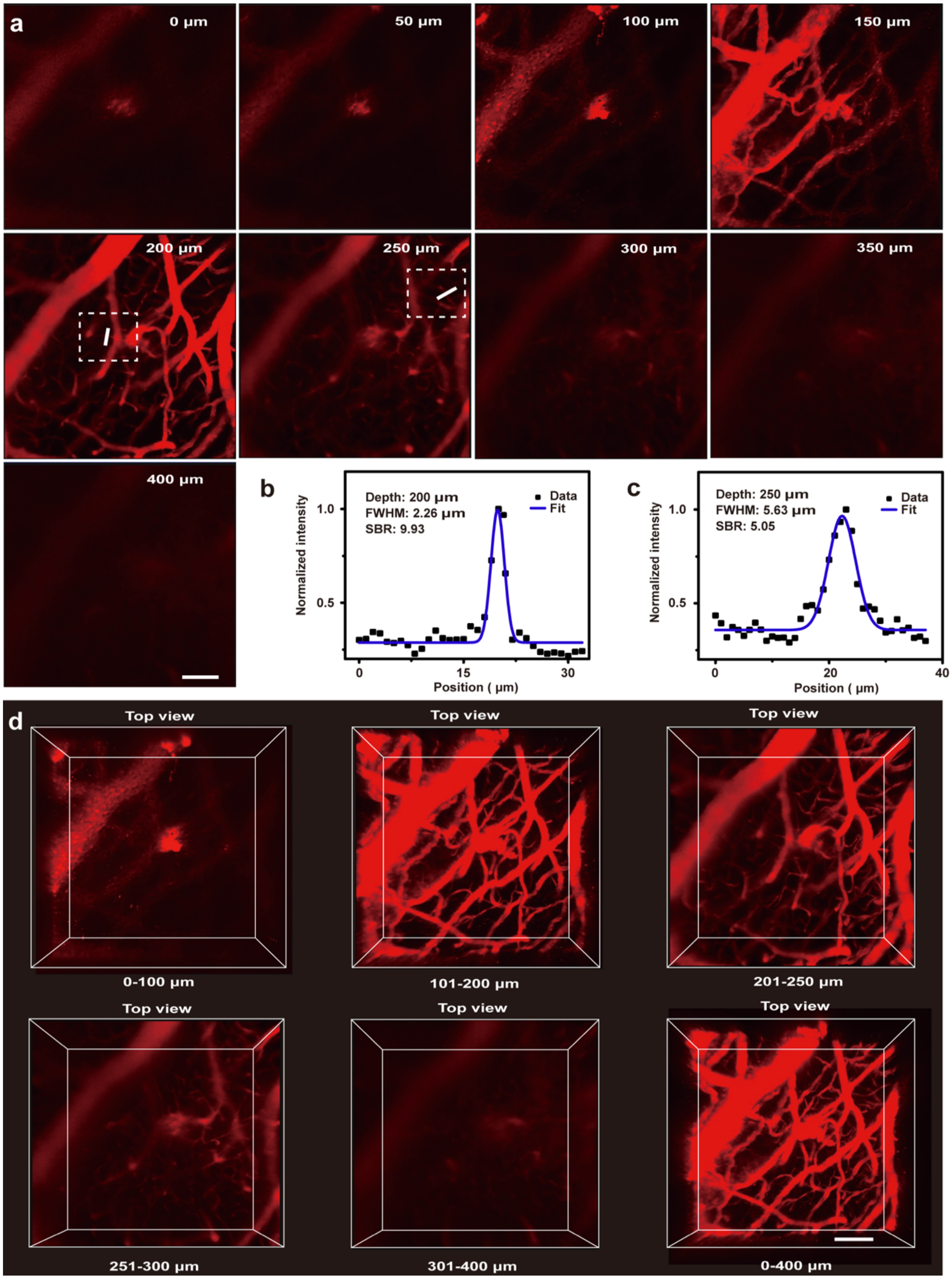

Figure S42. Intravital TPF imaging of mice cerebrovasculature with intact skull. (a) TPF images of the blood vessels at various vertical depths, 0-400 μm. Values of FWHM and SBR analysis of TPF images at (b) 200 and (c) 250 μm. (d) 3D reconstruction images of the

cerebrovasculature, 0-400 μm. The white dotted square on each image indicates the selected region of the fluorescence intensity profile. Ex: 960 nm. Scale bar: 100 μm.

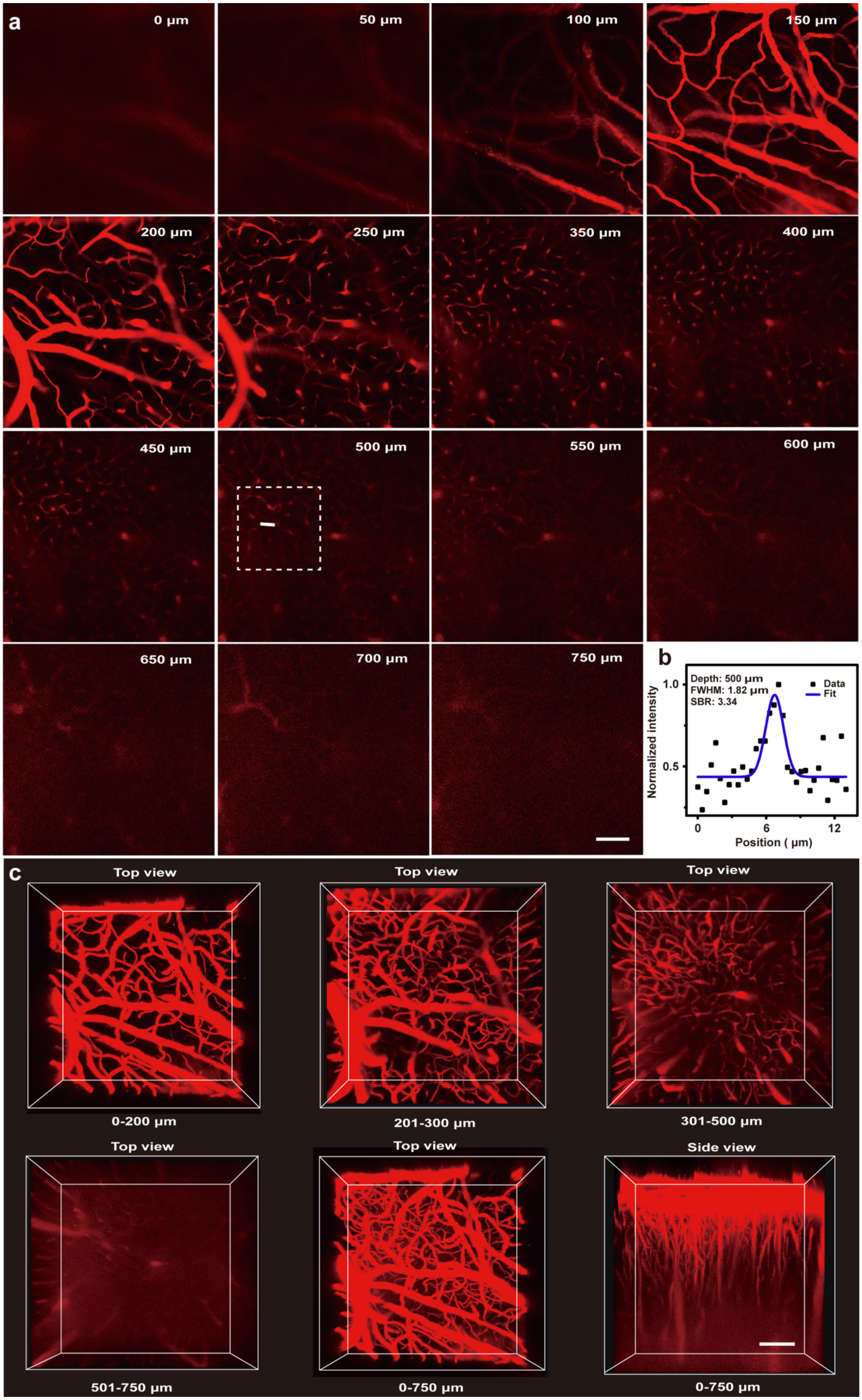

a
0 μm
50 μm
100 μm
150 μm
200 μm
250 μm
350 μm
400 μm
450 μm
500 μm
550 μm
600 μm
650 μm
700 μm
750 μm
b
Depth: 500 μm
FWHM: 1.82 μm
SBR: 3.34
Data
Fit
Normalized intensity
Position (μm)
c
Top view
0-200 μm
201-300 μm
301-500 μm
501-750 μm
0-750 μm
Side view

Figure S43. Intravital TPF imaging of mice cerebrovasculature with skull-thinning. (a) TPF images of the blood vessels at various vertical depths, 0-750 μm. (b) Values of FWHM and SBR analysis of TPF images at 500 μm. (c) 3D reconstruction images of the cerebrovasculature, 0-750 μm. The white dotted square on each image indicates the selected region of the fluorescence intensity profile. Ex: 960 nm. Scale bar: 100 μm.

Table S1.

Hematological parameters of the mice untreated, treated with TBF-24 at day 1, 8, 15, and the reference ranges of normal mice.

Parameters	Day	Control	TBF-24	Reference
ALP (U L^{-1})	1	226.75 ±6.29	233.59 ±23.19	22.52-474.35
	8	325.69 ± 15.21	288.92 ±24.61	
	14	260.20 ± 18.77	300.30 ±24.13	
ALT (U L^{-1})	1	63.25 ± 7.74	61.65 ± 7.32	10.06-96.47
	8	80.05 ± 6.70	65.11 ± 2.46	
	14	65.89 ± 8.11	61.39 ± 5.51	
AST (U L^{-1})	1	149.32 ± 16.01	152.70 ± 15.35	36.31-235.48
	8	204.06 ± 23.76	187.72 ± 5.45	
	14	189.79 ± 24.86	191.82 ±35.01	
CREA (μM)	1	19.46 ± 0.92	22.08 ± 3.97	10.91-85.09
	8	29.58 ± 1.76	23.75 ± 1.06	
	14	22.68 ± 1.46	27.52 ± 2.53	
BUN (mg dL^{-1})	1	20.54 ± 1.21	20.75 ± 0.73	10.81-34.74
	8	27.50 ± 2.05	27.10 ± 1.182	
	14	20.84 ± 2.05	26.01 ± 0.60	
RBC (10^{12} L^{-1})	1	8.94 ± 0.33	8.00 ± 0.39	6.36-9.42
	8	7.90 ± 0.67	8.28 ± 0.33	
	14	7.96 ± 0.45	8.49 ± 0.59	
HCT (%)	1	40.38 ± 0.25	40.95 ± 2.42	34.6-44.6
	8	40.85 ±2.13	39.88 ± 1.67	
	14	37.80 ± 1.88	40.0 ± 2.24	
HGB (g L^{-1})	1	135.25 ± 2.94	128.75 ± 4.92	110-143
	8	125.75 ± 6.94	126.75 ± 5.67	
	14	127.0 ± 2.45	123.50 ± 1.50	
PLT (10^9 L^{-1})	1	647.25 ± 100.71	671.75 ±7.60	450-1590
	8	923.50 ±139.85	814.50 ± 300.66	
	14	801.50 ± 100.85	949.25 ± 204.36	
MPV (fL)	1	5.48 ± 0.29	5.03 ± 0.13	3.8-6.0
	8	5.40 ± 0.23	5.18 ± 0.29	
	14	5.10 ± 0.35	5.40 ± 0.19	
MCV (fL)	1	49.98 ± 0.64	49.75 ± 0.74	48.2-58.3
	8	51.33 ± 0.49	50.85 ± 0.67	
	14	50.53 ± 0.68	51.05 ± 0.38	
MCHC (g L^{-1})	1	310.0 ± 4.18	306.0 ± 2.0	302-353
	8	312.25 ± 5.12	307.50 ± 1.12	
	14	306.0 ± 2.12	307.5 ±4.72	

Table S2.

A summary of recent intravital vascular TPF imaging parameters through an opened cranial window.

Probes	λex/λem (nm)	QY (%)	TPABCS (GM)	Injection dose	SBR	Resolution (μm)	Depth (μm)	Ref
AIEgen-Protein	840/ 640	12	110	10 mg kg^{-1}	58	1.05	656	[3]
Dyes	800/-	-	-	1 mM	-	-	500	[4]
PFBT loaded NPs	810/ 545	75	1085	0.4 μmol	-	-	500	[5]
BTPETQ dots	1200/ 700	19	7.63×10^{4}	0.05 mg	120	1.2 (900 μm)	924*	[6]
AIE NPs	1040/ 620	5	2.9×10^{6}	0.25 mg	-	-	700*	[7]
AIE NPs	820/ 598	32	3.43×10^{5}	22.5 pmol	-	-	100	[8]
AIE NPs	900/ 630	38.5	310	0.01 mg	-	4 (110 μm)	350	[9]
AIE NPs	1040/ 800	15.6	207	0.2 mg	-	1.8 (600 μm)	700*	[10]
AIE NPs	1040/ 620	6	3×10^{3}	0.87 pmol	-	1.92	800*	[11]
Polymer dots	810/ 650	57	8500 g	2.1 mg kg^{-1}	-	-	720	[12]
TQ BPN NPs	1300/810	13.9	1.22×10^{3}	0.2 mg	33 (150 μm)	4.9	1065*	[13]
CPdots	1200/ 725	20.6	1.21×10^{3}	2 mg kg^{-1}	5 (400 μm)	3.27	1010*	[14]
Pyrene dye NPs	960/ 640	53	150	150 nmol	-	-	1500**	[15]
Alexa680-Dextran	1280/ 680	-	-	100 μL, 5% w/v	1 (1500 μm)	1.9 (1010 μm)	1610**	[16]
TBF-24	960/ 628	68.7	479	5 mg kg^{-1}	1.5 (1100 μm)	2.7 (1100 μm)	1100	This Work

* Excited at NIR-II[6, 10, 13-14]; **High excitation power, i.e., maximal output power: up to 1.2 W and approximately 120 mW[15-16].

Reference:

[1] a) W. Wu, D. Mao, S. Xu, S. Ji, F. Hu, D. Ding, D. Kong, B. Liu, *Mater. Horiz.* **2017**, *4* (6), 1110; b) W. Wu, S. Xu, G. Qi, H. Zhu, F. Hu, Z. Liu, D. Zhang, B. Liu, *Angew. Chem. Int. Ed. Engl.* **2019**, *58* (10), 3062.
[2] W. J. Zhao, Z. K. He, J. W. Y. Lam, Q. Peng, H. Ma, Z. G. Shuai, G. X. Bai, J. H. Hao, B. Z. Tang, *Chem* **2016**, *1* (4), 592.
[3] S. Wang, F. Hu, Y. Pan, L. G. Ng, B. Liu, *Adv. Funct. Mater.* **2019**, *29* (29), 1902717.
[4] O. Garaschuk, R. I. Milos, A. Konnerth, *Nat Protoc* **2006**, *1* (1), 380.
[5] J. Geng, C. C. Goh, N. Tomczak, J. Liu, R. Liu, L. Ma, L. G. Ng, G. G. Gurzadyan, B. Liu, *Chem. Mater.* **2014**, *26* (5), 1874.
[6] S. Wang, J. Liu, C. C. Goh, L. G. Ng, B. Liu, *Adv. Mater.* **2019**, *31* (44), e1904447.
[7] Y. Wang, R. Hu, W. Xi, F. Cai, S. Wang, Z. Zhu, R. Bai, J. Qian, *Biomed. Opt. Express* **2015**, *6* (10), 3783.
[8] B. Chen, G. Feng, B. He, C. Goh, S. Xu, G. Ramos-Ortiz, L. Aparicio-Ixta, J. Zhou, L. Ng, Z. Zhao, B. Liu, B. Z. Tang, *Small* **2016**, *12* (6), 782.
[9] W. Qin, P. Zhang, H. Li, J. W. Y. Lam, Y. Cai, R. T. K. Kwok, J. Qian, W. Zheng, B. Z. Tang, *Chem. Sci.* **2018**, *9* (10), 2705.
[10] Y. Li, S. Liu, H. Ni, H. Zhang, H. Zhang, C. Chuah, C. Ma, K. S. Wong, J. W. Y. Lam, R. T. K. Kwok, J. Qian, X. Lu, B. Z. Tang, *Angew. Chem. Int. Ed. Engl.* **2020**, *59* (31), 12822.
[11] S. Samanta, M. Huang, S. Li, Z. Yang, Y. He, Z. Gu, J. Zhang, D. Zhang, L. Liu, J. Qu, *Theranostics* **2021**, *11* (5), 2137.
[12] N. Alifu, Z. Sun, A. Zebibula, Z. Zhu, X. Zhao, C. Wu, Y. Wang, J. Qian, *Opt. Commun.* **2017**, *399*, 120.
[13] J. Qi, C. Sun, D. Li, H. Zhang, W. Yu, A. Zebibula, J. W. Y. Lam, W. Xi, L. Zhu, F. Cai, P. Wei, C. Zhu, R. T. K. Kwok, L. L. Streich, R. Prevedel, J. Qian, B. Z. Tang, *ACS Nano* **2018**, *12* (8), 7936.
[14] S. Wang, J. Liu, G. Feng, L. G. Ng, B. Liu, *Adv. Funct. Mater.* **2019**, *29* (15), 1808365.
[15] M. Takezaki, R. Kawakami, S. Onishi, Y. Suzuki, J. Kawamata, T. Imamura, S. Hadano, S. Watanabe, Y. Niko, *Adv. Funct. Mater.* **2021**, *31* (20), 2010698.
[16] D. Kobat, N. G. Horton, C. Xu, *J. Biomed. Opt.* **2011**, *16* (10), 106014.
